# Methyltransferase G9a promotes cervical cancer angiogenesis and decreases patient survival

**DOI:** 10.18632/oncotarget.19060

**Published:** 2017-07-07

**Authors:** Ruey-Jien Chen, Chia-Tung Shun, Men-Luh Yen, Chia-Hung Chou, Ming-Chieh Lin

**Affiliations:** ^1^ Department of Obstetrics and Gynecology, National Taiwan University, Taipei 100, Taiwan; ^2^ Department of Pathology, National Taiwan University, Taipei 100, Taiwan

**Keywords:** G9a, angiogenesis, cancer cell proliferation, xenograft, patient survival

## Abstract

Research suggests that the epigenetic regulator G9a, a H3K9 histone methyltransferase, is involved in cancer invasion and metastasis. Here we show that G9a is linked to cancer angiogenesis and poor patient survival. Invasive cervical cancer has a higher G9a expression than cancer precursors or normal epithelium. Pharmacological inhibition and genetic silencing of G9a suppresses H3K9 methylation, cancer cell proliferation, angiogenesis, and cancer cell invasion/migration, but not apoptosis. Microarray and quantitative reverse transcription polymerase chain reaction analyses reveal that G9a induces a cohort of angiogenic factors that include angiogenin, interleukin-8, and C-X-C motif chemokine ligand 16. Depressing G9a by either pharmacological inhibitor or gene knock down significantly reduces angiogenic factor expression. Moreover, promoting G9a gene expression augments transcription and angiogenic function. A luciferase reporter assay suggests that knockdown of G9a inhibits transcriptional activation of interleukin-8. G9a depletion suppresses xenograft tumor growth in mouse model, which is linked to a decrease in microvessel density and proliferating cell nuclear antigen expression. Clinically, higher G9a expression correlates with poorer survival for cancer patients. For patients’ primary tumors a positive correlation between G9a expression and microvessel density also exists. In addition to increasing tumor cell proliferation, G9a promotes tumor angiogenesis and reduces the patient survival rate. G9a may possess great value for targeted therapies.

## INTRODUCTION

Cancer’s biological capabilities are often listed as including unlimited cell proliferation [1], angiogenesis [2], telomere shortening [3], suppression of apoptosis [4], and invasion and metastasis [5]. Underlying these factors is genomic instability [6, 7]. In general, human tumors undergo a massive overall loss of DNA methylation and specific patterns of hypermethylation [8]. Cells lacking H3K9 methylation have displayed disorganized nucleoli and genomic instability [9]. DNA methylation with histone modification is a common hallmark of cancer cells [10, 11]. These epigenetic alterations are as important to cancer cell transformation as genetic mutations [12]. Moreover, a DNA methylation signature may identify cancer patients who might benefit from a more aggressive treatment [13].

Cervical cancer is a common solid tumor malignancy. A 2015 study reported that in 2012 there were an estimated 527,600 new cases and 265,700 deaths worldwide from cervical cancer [14]. Its pathogenesis is associated with oncogenic human papillomavirus (HPV) infection. During the progression of cervical cancer, HPV genomes and cellular tumor suppressor genes can become methylated [15–17]. The methylation status of the HPV viral genome changes not only in the context of the viral life cycle but also in the context of neoplastic disease progression that results in cancer [18]. DNA methylation analysis is a useful tool for detecting cervical cancer and its precursors when doing a cytology test [19]. Increased DNA methylation has even been recommended as a potential biomarker for precancerous cervical disease [20].

G9a is a mammalian H3K9 histone methyltransferase which catalyzes the methylation of H3K9 and contributes to the epigenetic silencing of tumor suppressor genes. Emerging evidence suggests that G9a is required to maintain a malignant phenotype [21]. G9a’s activity can be pharmacologically inhibited by BIX01294 [22]. Depression of G9a may induce the oncogenic transformation of immortalized primary human cells [23]. It has been suggested that modifying H3K9 methylation may promote invasiveness and metastasis in some cancer cells [24]. G9a is required for transcriptional action in order to sustain cancer cell proliferation [25, 26]. G9a depletion also potentiates cell death in cancer cells [27]. A positive correlation between G9a and p53 expression is associated with better survival for lung cancer patients [28]. However, whether G9a is expressed in cervical cancer and cancer precursors is not known. In this study, we investigated whether G9a is tumorigenic in cervical cancer and if it correlates with clinical survival.

## RESULTS

### High G9a expression in cervical cancer

**Figure 1:**
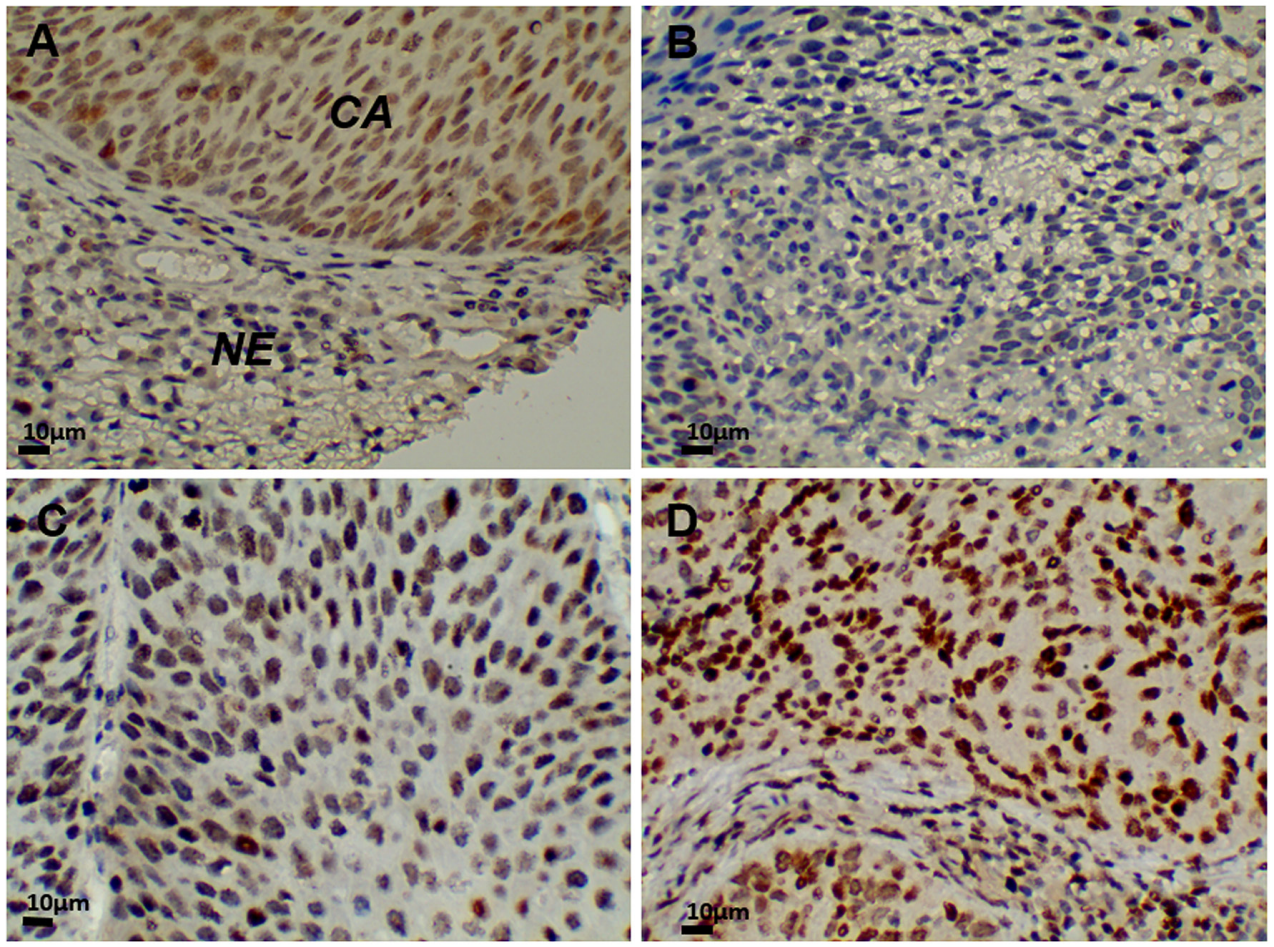
G9a expression in cervical cancer tissue (400x) **(A)** In normal squamous epithelium of the exocervix, G9a was not expressed. Strong immunostaining detected G9a in squamous cell carcinoma nuclei. **(B)** G9a positive grade 1. **(C)** G9a positive grade 2. **(D)** G9a positive grade 3. CA: carcinoma. NE: normal cervical epithelium.

First, we evaluated G9A expression in cervical cancer. We examined G9A expression in sections from normal and diseased uterine cervices using immunohistochemical staining. G9A expression was detected in the nuclei of carcinoma cells (Figure 1A). Positive nuclear staining was marked by the color brown, while counter staining was expressed by blue. A higher expression of G9A was found in carcinoma cells than in the nuclei of cancer precursors or in normal epithelium. A positive expression level of G9A was scored as grades 1, 2 or 3 (Figure 1B-1D). For expression levels, a higher grade was associated with a higher staining intensity.

A total of 443 cases were studied. Of the 321 tissue array samples, 26 were of a normal cervix, 109 were of cancer precursors, and 186 were of invasive cervical carcinoma. Normal cervical specimens obtained surgically from 122 patients with benign disease were also used in our study. For normal cervices, 16.2% (24/148) had a positive staining. For cancer precursors, 33 of 109 (30.3%) had a positive staining, while 117 (62.9%) of 186 samples from invasive carcinomas had a positive staining. Cervical carcinoma had the highest G9a expression (Table 1). No statistical difference was found between cancer stages I and II, between squamous cell carcinoma and adenocarcinoma, or between tumor cell differentiations. Together, Figure 1 and Table 1 show that G9a has a high expression level in cancer cells.

**Table 1:** G9a expression in epithelial cells of normal cervix, cancer precursors and invasive carcinoma of the uterine cervix

Characteristics	Case no. or mean ± SD	G9a (%)				*p*-value
		Negative	Positive			
			1	2	3	
Total cases	443	269 (60.7%)	80 (18.1%)	60 (13.5%)	34 (7.7%)	
Age	46.2 ± 10.1	47.7 ± 9.9	43.9 ±10.4*	43.8 ± 9.9*	44.8 ± 9.9	0.003*
Histological diagnosis	< 0.0001^#,¶^					
Normal cervix	148	124 (83.8%)	19 (12.8%)	5 (3.4%)	0	
Cancer precursors	109	76 (69.7%)	27 (24.8%)	6 (5.5%)	0	
Invasive carcinoma	186	69 (37.1%)	34 (18.3%)	49 (26.3%)	34 (18.3%)	
Tumor stage^†^	*NS*^#,*¶*^					
Stage I	161	58 (36.0%)	30 (18.6%)	43 (26.7%)	30 (18.6%)	
Stage II	25	11 (44.0%)	4 (16.0%)	6 (24.0%)	4 (16.0%)	
Tumor cell type^†^	*NS*^##,¶^					
Squamous cell carcinoma	151	54 (35.8%)	28 (18.5%)	39 (25.8%)	30 (19.9%)	
Adenocarcinoma	35	15 (42.9%)	6 (17.1%)	10 (28.6%)	4 (11.4%)	
Tumor cell differentiation^†,§^	*NS*^#,*¶*^					
Well differentiated	12	2 (16.7%)	2 (16.7%)	4 (33.3%)	4 (33.3%)	
Moderately differentiated	67	26 (38.8%)	9 (13.4%)	23 (34.3%)	9 (13.4%)	
Poorly differentiated	94	35 (37.2%)	21 (22.3%)	21 (22.3%)	17 (18.1%)	

*ANOVA with *post hoc* Dunnett’s test compared to G9a negative. ^#^Chi-square test for trend. ^¶^Chi-square test after regrouping G9a as negative and positive expressions. ^†^invasive carcinoma. ^##^Chi-square test. ^§^information not provided in 13 cases. *NS*: non-significant.

### Suppression of G9a represses cervical cancer cell proliferation

**Figure 2:**
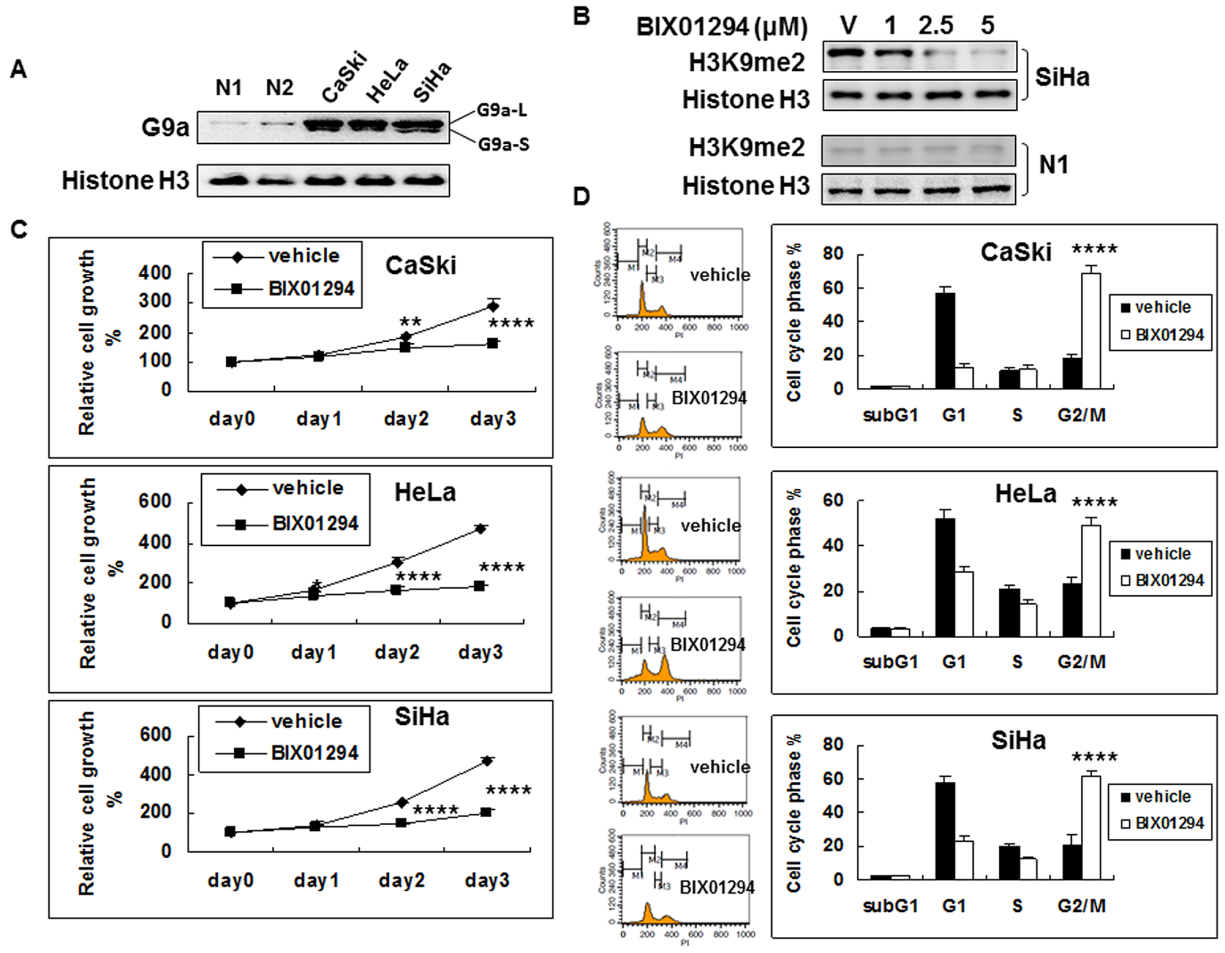
G9a expression in cervical cancer cells and the anti-cell proliferation effect of G9a chemical inhibitor BIX01294 **(A)** Nuclear protein (10 μg) from the exponential phase of cervical cancer or normal human cervical epithelial cells (N1 and N2; 2 different lots) were used for G9a determination by western blot. Histone H3 was used as a loading control. L: long isoform. S: short isoform. **(B)** Effect of G9a chemical inhibitor BIX01294 on the methylation of histone 3 lysine 9. Histone H3 was used as a loading control. v: vehicle. The BIX01294 concentrations were 0 (vehicle), 1, 2.5, and 5 μM, respectively. **(C)** Effect of BIX01294 on the cell growth of cervical cancer cell lines. Cells were treated with 5 μM of BIX01294 at different times: viable cells were determined by MTT assay. Relative cell growth rates from day 0 were calculated. *n* = 5, *p < 0.05; **p < 0.01;****p < 0.0001. Data are presented as mean ± SD. **(D)** Effect of BIX01294 on the cell cycle of cervical cancer cell lines. Cells were treated with 5 μM of BIX01294: after 3 days, cell cycle was determined by propidium iodide staining. Different cell cycle phases are quantified. *n* = 5, ****p < 0.0001. Data are presented as mean ± SD.

We used western blot on cervical cancer cell lines (SiHa, HeLa, and CaSki) and on normal human cervical epithelial cells (primary culture from Cell Application, Inc., San Diego) to determine G9a expression. G9a is strongly expressed in three cervical cancer cell lines, but its expression in normal human cervical epithelial cells is not pronounced (Figure 2A). We then studied the effect of G9a chemical inhibitor BIX01294 on the methylation of histone 3 lysine 9. The BIX01294 concentrations were 0 (v: vehicle), 1, 2.5, and 5 μM, respectively. Without BIX01294 treatment, H3K9me2 was highly expressed in cancer cells but was expressed at a much lower level in normal epithelial cells (Supplementary Figure 1). After treatment with BIX01294, both 2.5 μM and 5 μM of BIX01294 significantly lowered bulk H3K9me2 levels in cancer cells, while in normal epithelial cells H3K9me2 expression remained low after BIX01294 treatment. Histone H3 was used as a loading control. BIX01294 effectively inhibited H3K9me2 using doses of 2.5 and 5 μM (Figure 2B, Supplementary Figure 2). Immunohistochemical staining in a tissue array also showed that in contrast to a high level of H3K9me2 in the cancer cells, the expression level in normal cells was low (Supplementary Figure 3). Overall, cervical cancer has a high level of H3K9me2 expression which can be inhibited by BIX01294 in a dose dependent manner. Normal cervix epithelial cells have a low level of H3K9me2 expression, and accordingly BIX01294 inhibition is not pronounced. Consequently, a BIX01294 concentration of 5 μM was used for the experiments that followed (except for a 2 μM BIX01294 concentration that was chosen for chick embryo chorioallantoic membrane (CAM) assay).

We further investigated the effect of G9a on the growth of cancer cells by treating SiHa, HeLa and CaSki cells with vehicle or with BIX01294 for 1 to 3 days. Relative cell growth rates were determined using a 3-(4,5-dimethylthiazol-2-yl)-2,5-diphenyl tetrazolium bromide (MTT) cell proliferation assay for cellular viability and activity (Figure 2C). Results revealed that BIX01294 significantly inhibited cancer cell proliferation by day 2 or 3 in all three cervical cancer cell lines. Normal cervix epithelial cells are not proliferative (Supplementary Figure 4). To clarify whether the cell growth retardation effect was due to cell proliferation inhibition or to cytotoxic induction, SiHa, HeLa and CaSki cells that were treated with vehicle or with BIX01294 for 72 hrs were subjected to flow cytometry for cell cycle analysis (Figure 2D). Results revealed that treatment with BIX01294 caused significant cell cycle G2/M arrest without increasing the subG1 fraction. Overall, Figure 2 shows that cancer cells express G9a, that BIX01294 may inhibit G9a bioactivity (H3K9me2 expression), and that BIX01294 inhibits cancer cell proliferation but does not induce cancer cell apoptosis.

**Figure 3:**
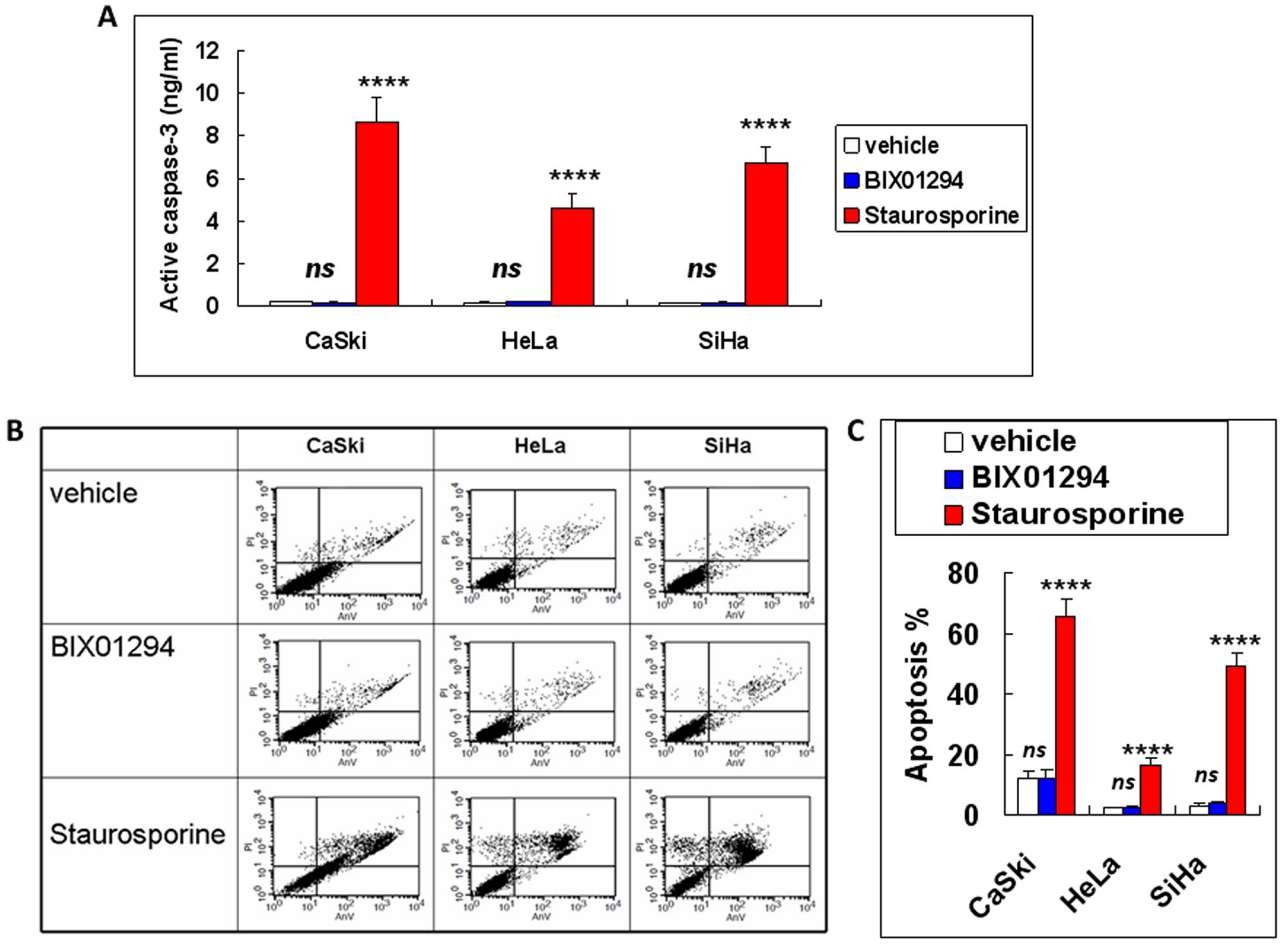
Apoptotic effect of BIX01294 on cervical cancer cells Cervical cancer cells (SiHa, HeLa and CaSki) were treated with BIX01294 for three days. Cervical cancer cells treated with 10 μg of staurosporine for 3 hrs were used as a positive control. **(A)** Active caspase-3 assay. Data presented are the level of active caspase-3. *n* = 3. ****p < 0.0001 (staurosporine *vs.* vehicle). *ns*: non-significant (BIX01294 *vs*. vehicle). **(B)** Flow cytometric assay by annexin-V/propidium iodide double staining for cancer cells after different treatment. **(C)** Quantitative data from Figure 3B. Apoptosis % were a sum of early apoptosis (lower right quadrant of cytogram, annexin-V positive and propidium iodide negative) and late apoptosis (upper right quadrant, annexin-V positive and propidium iodide positive). *n* = 3. ****p < 0.0001 (staurosporine *vs.* vehicle). *ns*: non-significant (BIX01294 *vs*. vehicle). AnV: annexin-V. PI: propidium iodide.

An active caspase-3 assay and annexin V/propidium iodide (PI) double staining were then performed; they showed that BIX01294 treatment at a dose of 5 μM does not cause cancer cell apoptosis (Figure 3A, 3B & 3C). Also, the cell cycle arrest for BIX01294-treated cancer cells noted in Figure 2D was not due to BIX01294-induced apoptosis. Overall, Figures 2 & 3 suggest that while G9a promotes cancer cell proliferation it does not influence cancer cell apoptosis.

### G9a promotes angiogenic factor expression

In cervical cancer, a decrease in tumor growth has been linked with decreased angiogenesis [29]. Angiogenesis has also been tied to the tumorigenesis, clinical diagnosis and management of cervical cancer [30]. Notably, combining angiogenesis blockade with other chemotherapy agents has been suggested for the treatment of persistent, recurrent, and metastatic cervical cancer [31]. Anti-apoptosis is another important property of cervical cancer cells [32]. Accordingly, in order to address a possible molecular mechanism for G9a in cervical cancer cells we studied both the angiogenic and apoptotic effects of G9a.

**Figure 4:**
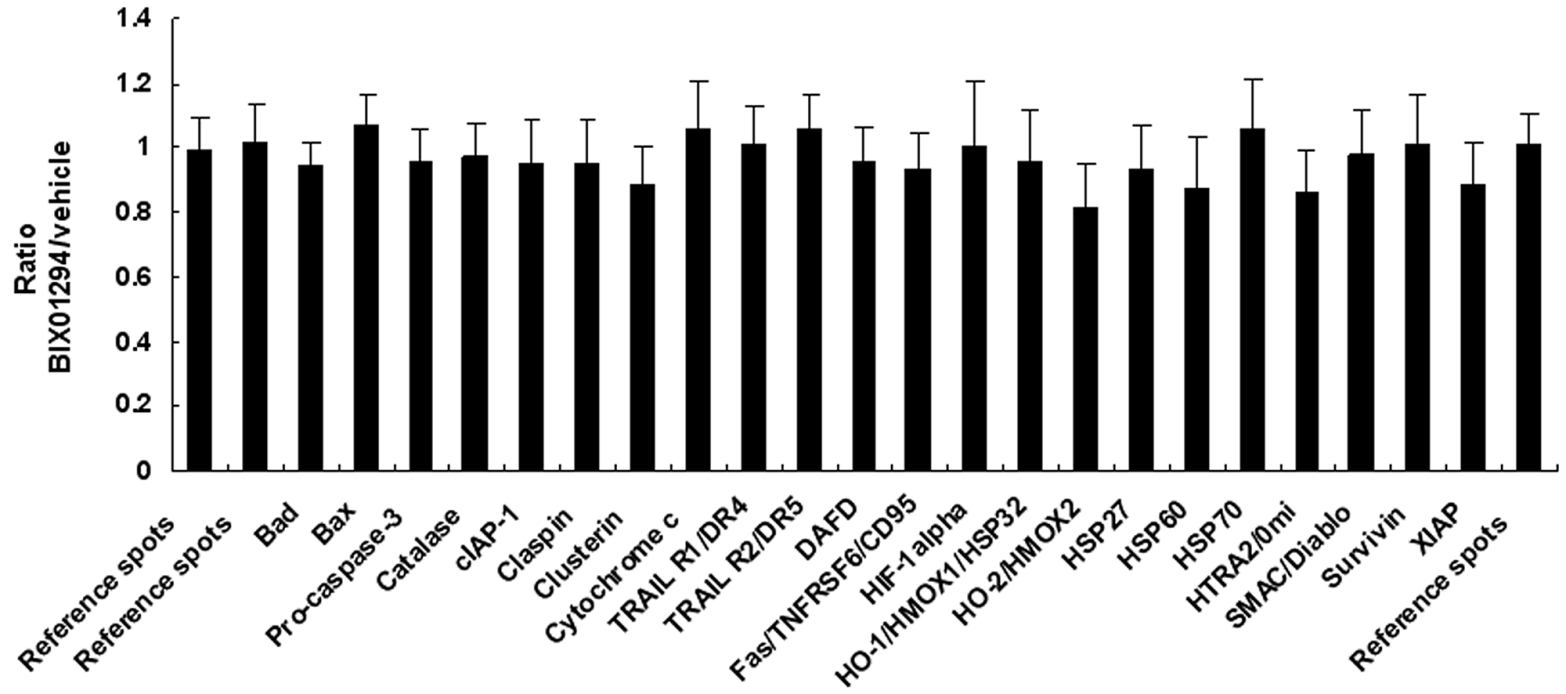
Quantitative results from effect of BIX01294 on apoptosis-related protein expression SiHa cells were treated with BIX01294 (5 μM) for 4 hrs; after washing out the medium, cells were incubated in fresh culture medium for 24 hrs to collect conditioned medium. Total cell lysate was used for apoptosis-related protein expression pattern analysis. Quantitative results are of apoptosis-related protein expression in SiHa cells. No statistical difference was found between the apoptosis proteins and the reference spots. Data are presented as mean ± SD.

**Figure 5:**
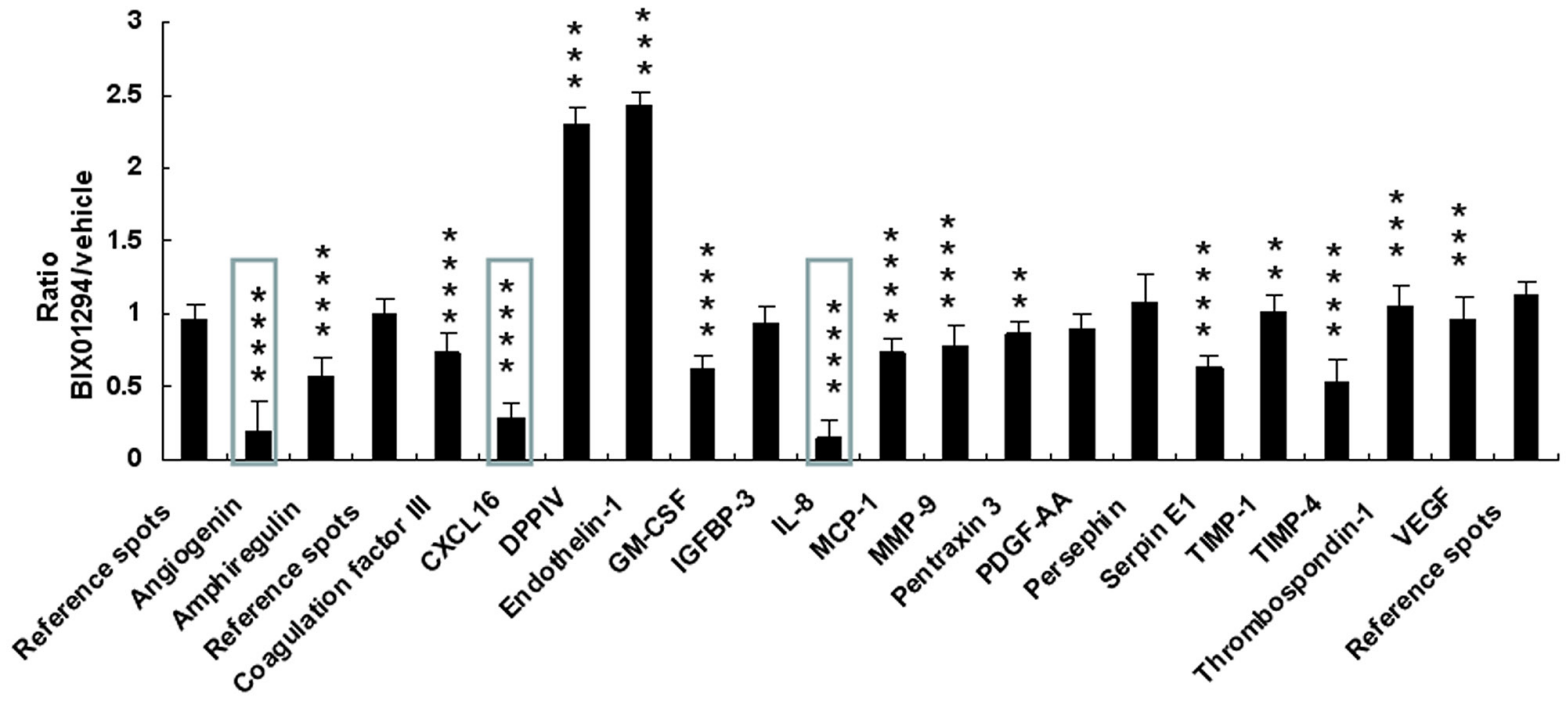
Quantitative results from effect of BIX01294 on angiogenesis-related protein expression SiHa cells were treated with BIX01294 (5 μM) for 4 hrs; after washing out the medium, cells were incubated in fresh culture medium for 24 hrs to collect conditioned medium. Conditioned medium was used for angiogenic factor analysis. Quantitative results are of angiogenic factor expression in SiHa cells (**p < 0.01; ***p < 0.001; ****p < 0.0001). IL-8: interleukin-8. Data are presented as mean ± SD. (BIX01294, *n* = 3; control, *n* = 3). Three proteins shown in box had a BIX01294/vehicle ratio of less than 0.5. They were used for gene knockdown and overexpression studies.

SiHa cells were treated with BIX01294 for 24 hrs, after which the cell lysate was used for apoptosis-related protein assays. These assays revealed that although 22 apoptotic proteins were expressed (Supplementary Figure 5, Supplementary Table 1), this expression was not changed by BIX01294 treatment (Figure 4). Conditioned medium was used for angiogenesis-related protein expression pattern analysis (Supplementary Figure 6, Supplementary Table 2).Quantitative results revealed that BIX01294 treatment significantly influenced most angiogenic factor expressions (Figure 5). Among the 19 expressed angiogenic factors, 14 factors (including angiogenin, amphiregulin, coagulation factor III, C-X-C motif chemokine ligand 16 (CXCL16), GM-CSF, interleukin-8, MCP-1, MMP-9, pentraxin 3, serpin E1, TIMP-1, TIMP-4, thrombospondin-1 and VEGF) were significantly inhibited, two factors (DPPIV and endothelin-1) were found to be induced by BIX01294, and three factors were not changed (IGFBP-3, PDGF-AA and persephin).

In order to clarify which angiogenic factors influence the effect angiogenesis has on proliferation, conditioned medium from vehicle or BIX01294-treated cancer cells was used to culture their own cancer cells. Our results revealed that angiogenic factors did not significantly influence their own cell proliferation (Supplementary Figure 7).

**Figure 6:**
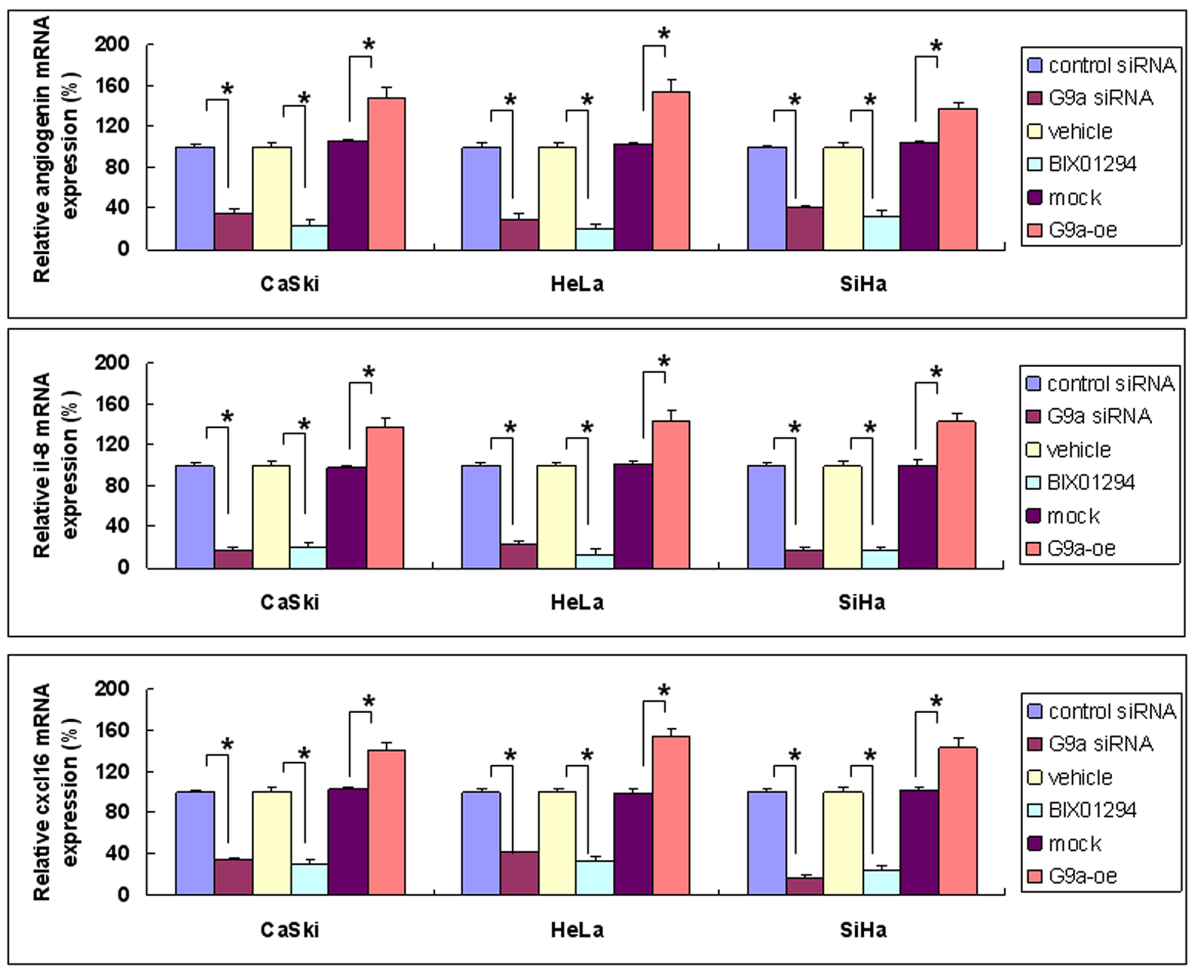
Angiogenic factors’ gene expression as determined by G9a gene silencing, pharmacologic inhibition of G9a, and G9a overexpression Cervical cancer cells (SiHa, HeLa and CaSki) were treated in one of the following ways: with control siRNA or G9a siRNA for 24 hrs; with BIX01294 (5 μM) or vehicle for 24 hrs; or with transient transfection with control plasmid (mock) or G9a over-expression plasmid (G9a-oe) for 48 hrs. The angiogenin, interleukin-8 (il-8) or cxcl16 mRNA expression was determined by real-time quantitative RT-PCR. Data presented here are the relative percentages of induction. Data are compared between the indicated groups. *p < 0.05, *n* = 3.

**Figure 7:**
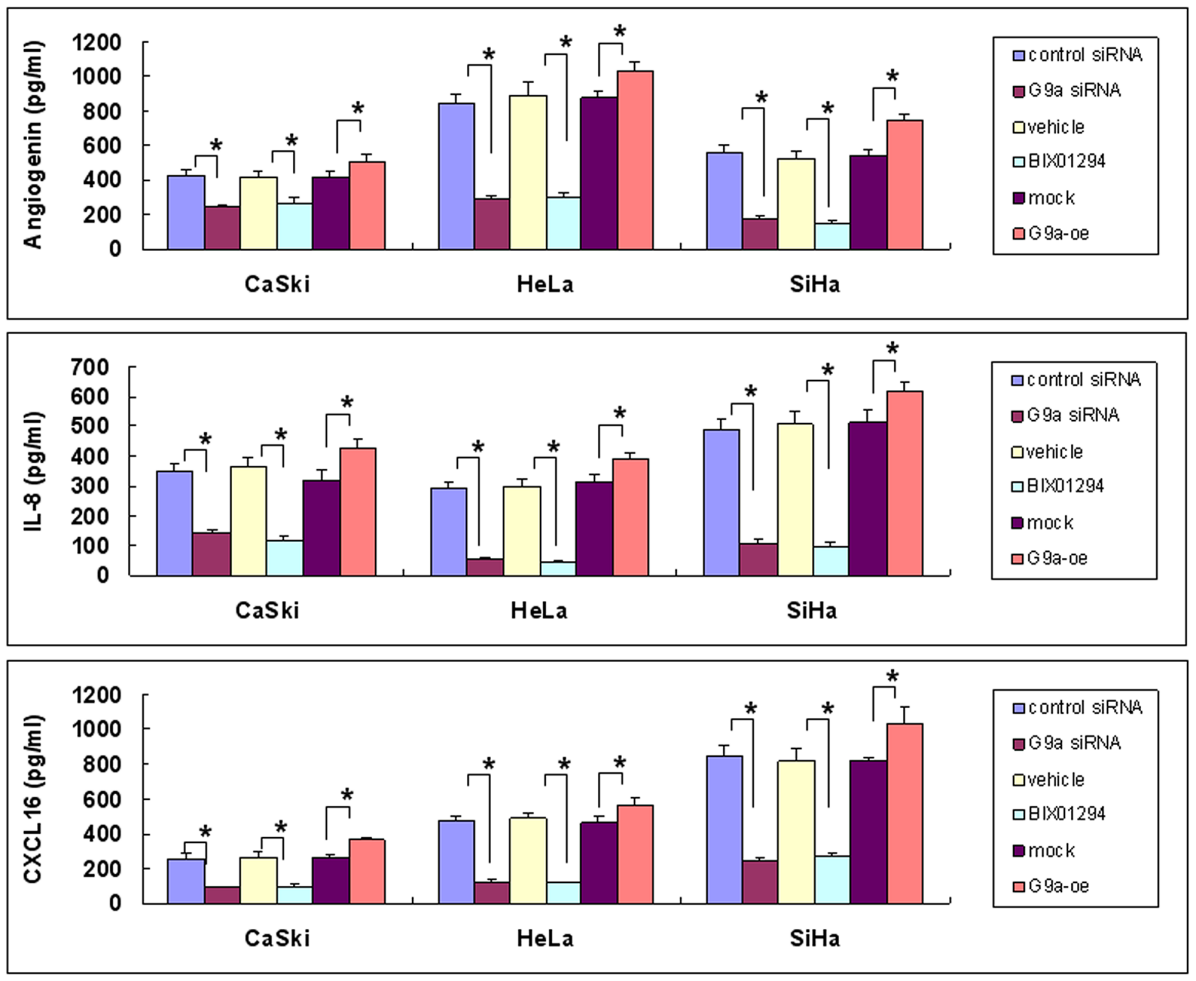
Angiogenic protein expression as determined by G9a gene silencing, pharmacologic inhibition of G9a, and G9a overexpression Cervical cancer cells (SiHa, HeLa and CaSki) were treated in one of the following ways: with control siRNA or G9a siRNA for 24 hrs; with BIX01294 (5 μM) or vehicle for 24 hrs; or with transient transfection with control plasmid (mock) or G9a over-expression plasmid (G9a-oe) for 48 hrs. The expression of angiogenin, interleukin-8 (IL-8) or CXCL16 protein in a 24 hr-conditioned medium was determined by EIA. Data are compared between the indicated groups. *p < 0.05, *n* = 3.

Image-J software was used for array quantification of angiogenic factor expression. Three such factors with a BIX01294/vehicle ratio of less than 0.5 (angiogenin, interleukin-8, and CXCL16) were selected for further study (cf. Figure 5). G9a gene knockdown repressed both mRNA and protein expression, while over-expression of G9a promoted both mRNA and protein expression (Figures 6 & 7). Quantitative PCR data on G9a mRNA (Supplementary Figure 8A) and G9a protein levels (Supplementary Figure 8B) showed that G9a gene knockdown efficiently repressed its transcription and translation. A luciferase assay revealed that knockdown of G9a inhibited transcriptional regulation of interleukin-8 (Supplementary Figure 9).

A Western blot assay of three apoptotic proteins (cIAP-1, HSP70, and XIAP) revealed that neither inhibition of G9a by BIX01294 nor G9a gene knockdown induces apoptosis (Supplementary Figure 10). These results reveal that depression of G9a by either pharmacological inhibitor or gene knock down significantly reduces angiogenic factor expression, while overexpression of G9a increases angiogenic factor expression.

### Suppression of G9a decreases angiogenesis *in vitro*

**Figure 8:**
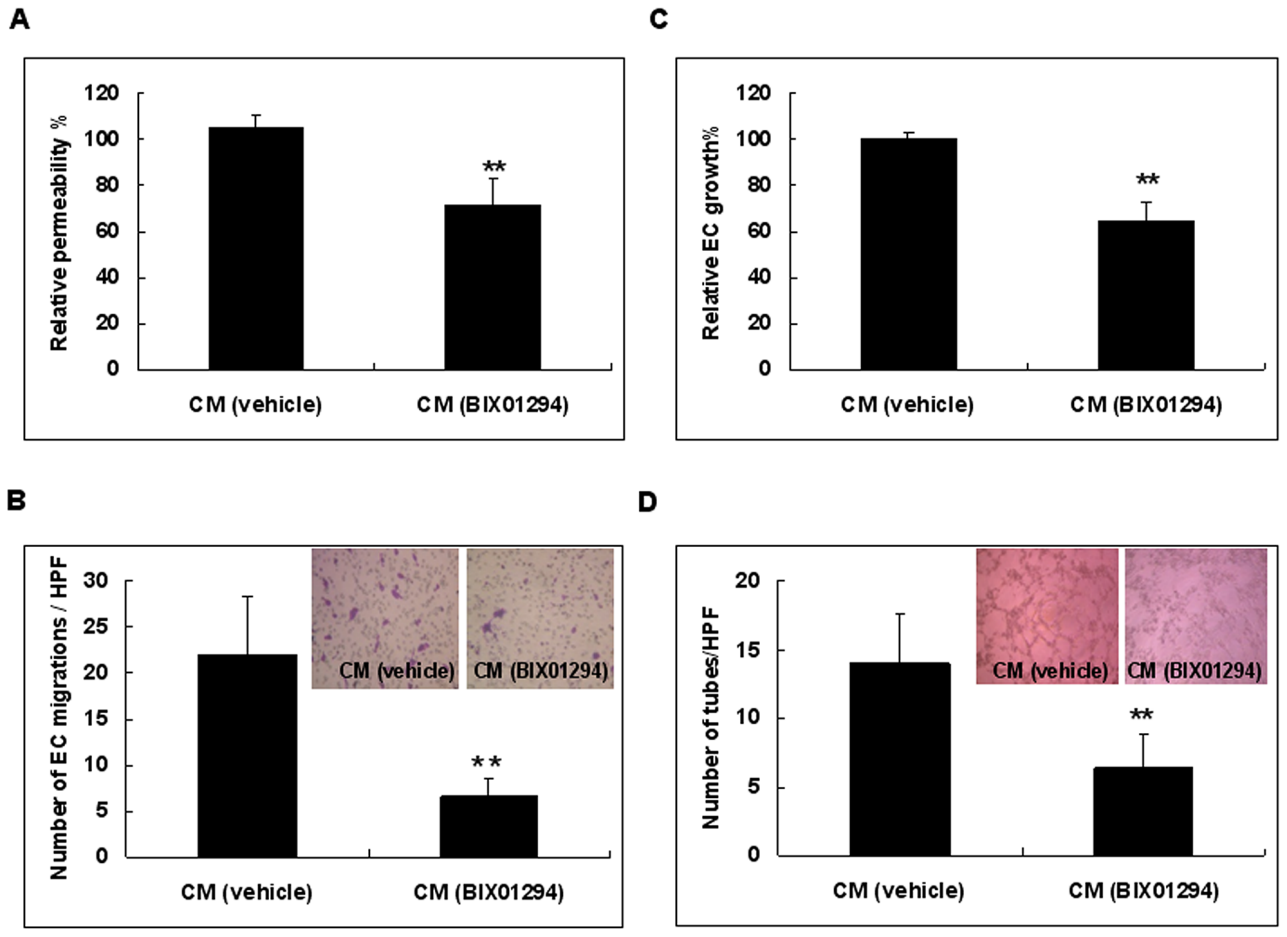
Cervical cancer cells treated with conditioned medium from BIX01294 lose angiogenic capability SiHa cells were treated with BIX01294 (5 μM) for 4 hrs; after washing out the medium, cells were incubated in fresh culture medium for 24 hrs to collect conditioned medium. Conditioned medium was used for the following *in vitro* angiogenesis assays: **(A)** Conditioned medium from vehicle or BIX01294 treated cells was used for an endothelial cell permeability assay. Data were the relative permeability, in which vehicle-treated conditioned medium is defined as 100%. *n* = 5. **p < 0.01. **(B)** Endothelial cell migration assay. Data were the number of migrated endothelial cells per HPF (100x) under different conditions. *n* = 5. **p < 0.01. **(C)** Conditioned medium from vehicle or BIX01294 treated cells was used for an endothelial cell proliferation assay. Data were the relative endothelial cell growth percentages under different conditions, in which vehicle-treated conditioned medium is defined as 100%. *n* = 5. **p < 0.01. **(D)** Endothelial cell tube formation assay. Data were the number of polygonal vascular tube formations per HPF (100x) under different conditions. *n* = 5. **p < 0.01. Data are presented as mean ± SD. CM: conditioned medium. EC: endothelial cell. HPF: high power field. veh: vehicle.

We further investigated the net effect of BIX01294 on the angiogenic capability of SiHa cells *in vitro*. Conditioned medium from BIX01294 or vehicle treated SiHa cells was used for angiogenesis assays. Our results revealed that, relative to vehicle treated cells, conditioned medium from BIX01294 treated cells significantly reduced endothelial cell permeability (Figure 8A). Using an endothelial cell migration assay, we found that conditioned medium from treated cells significantly reduced endothelial cell migration when compared to vehicle treated cells (Figure 8B). Meanwhile, conditioned medium from BIX01294 treated cells significantly reduced endothelial cell proliferation when compared to vehicle treated cells (Figure 8C). Furthermore, conditioned medium from BIX01294 treated cells significantly reduced the number of polygonal vascular tube formations of endothelial cells relative to vehicle treated cells (Figure 8D). For HeLa and CaSki cells, treatment with BIX01294 decreased angiogenesis (Supplementary Figure 11). Antibodies specific to angiogenin, interleukin-8, and CXCL16 also depressed polygonal vascular tube formation (Supplementary Figure 12). These *in vitro* angiogenesis assays suggest that suppression of G9a has a net anti-angiogenic effect. Collectively, Figures 4 to 8 reveal that the epigenetic regulator G9a promotes angiogenesis.

### G9a increases cervical cancer cell migration and invasion

**Figure 9:**
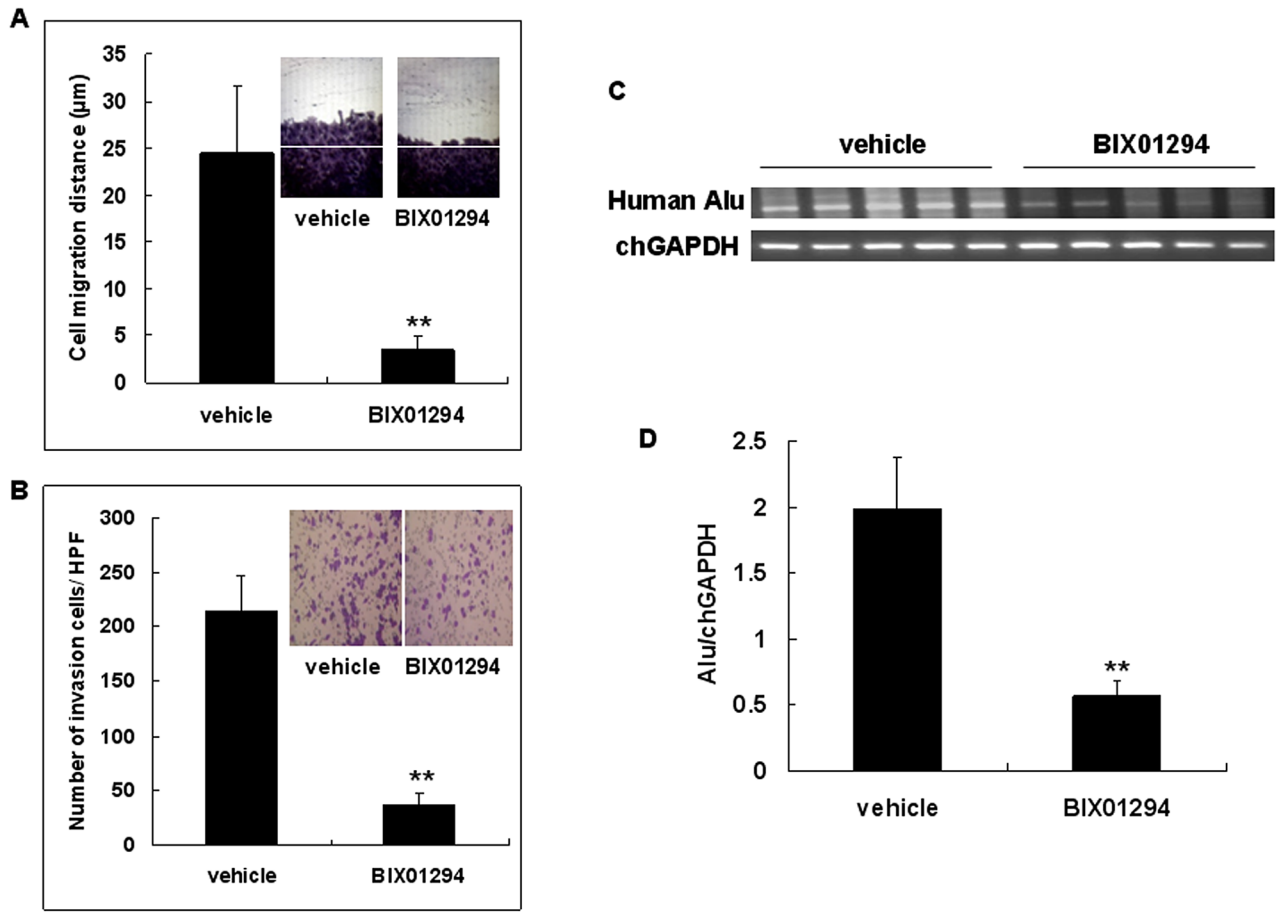
G9a inhibitor BIX01294 inhibits cervical cancer cell migration and invasion **(A)** Confluent SiHa cells were pretreated with 5 μM of BIX01294 24 hrs prior to an *in vitro* wound healing migration assay. Migration distances were measured in HPF (100x). *n* = 5. **p < 0.01. Data are presented as mean ± SD. **(B)** SiHa cells were pretreated with BIX01294 (5 μM) 24 hrs prior to an *in vitro* transwell invasion assay. Invaded cells were calculated in HPF (100x). Data are presented as mean ± SD. *n* = 5. **p < 0.01. **(C)** SiHa cells which were pretreated with vehicle or 2 μM of BIX01294 for 24 hrs were used to analyze the intravasation phenotype *in vivo* by chick embryo chorioallantoic membrane (CAM) assay. SiHa cells (1 x 10^6^) were inoculated on the CAM of 9-day-old chick embryos; the membrane at the opposite side of the egg was recovered after a 48 hr incubation. Invasion cells were determined by detecting human DNA with Alu sequences in each CAM sample by PCR; chick (Ch) GAPDH was used as an internal control. **(D)** Ratio of Alu to chGAPDH in vehicle and BIX01294 treated groups. *n* = 5. **p < 0.01. Data are presented as mean ± SD. HPF: high power field.

To address the effect of G9a on cervical cancer cell migration, confluent SiHa cells were pretreated with BIX01294 or vehicle 24 hrs prior to performing an *in vitro* wound healing migration assay (Figure 9A). SiHa cells were also pretreated with BIX01294 or vehicle 24 hrs prior to performing an *in vitro* transwell invasion assay. Results revealed that BIX01294 significantly reduced the number of invaded cells (Figure 9B). SiHa cells which were pretreated with BIX01294 or vehicle were used to evaluate whether G9a promotes cervical cancer cell invasiveness *in vivo*; this was done by analyzing the intravasation phenotype using an *in vivo* CAM assay. Invasive cells were determined by detecting human DNA with Alu sequences in each CAM sample by PCR. The intensity of human Alu PCR was found to be abundant in the vehicle group rather than in the BIX01294 groups (Figure 9C). These quantitative results demonstrate that the ratio of Alu to chick glyceraldehyde-3-phosphate dehydrogenase (chGAPDH) in the vehicle group was significantly higher than in the BIX01294 treated groups (Figure 9D). Taken together, our results from *in vitro* and *in vivo* cell migration/invasion assays (Figure 9) suggest that G9a promotes cervical cancer cell migration and invasion.

### G9a and xenograft tumor growth

**Figure 10:**
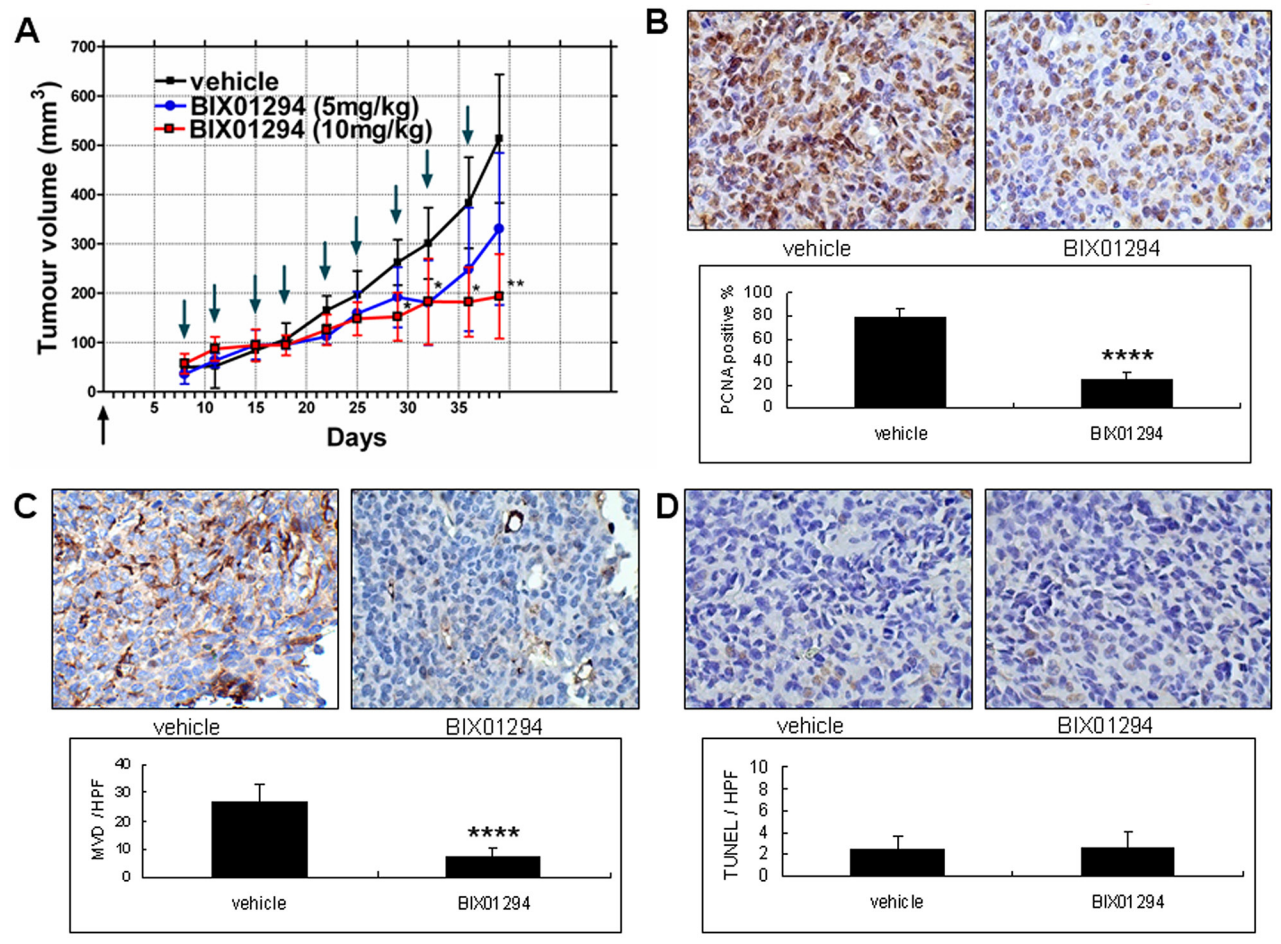
Effect of BIX01294 on the tumor growth curve of SiHa cells SiHa cell lines were seeded in the right hind limb of SCID mice. **(A)** The mice were divided randomly into three groups. In the first group, vehicle was injected intraperitoneally (normal saline, 100 μL, twice a week). For the second group, mice were injected intraperitoneally with BIX01294 at 5 mg/kg (mouse body weight; dissolved in normal saline, 100 μL, twice a week). Finally, in the third group mice were injected intraperitoneally with BIX01294 at 10 mg/kg (mouse body weight; dissolved in normal saline, 100 μL, twice a week). In the BIX01294 10mg/kg group, tumor growth was inhibited significantly 29 d after inoculation. In the BIX01294 5mg/kg group, tumor growth did not differ significantly from the control group (*n* = 10 in each group. *p < 0.05; **p < 0.01; ANOVA with *post hoc* Tukey’s test). Upward arrow **↑** (in black): cancer cell inoculation. Downward arrow **↓** (in green): vehicle or BIX01294 injection. **(B)** Tumors from vehicle and BIX01294 (10 mg/kg) treatment groups were harvested at the time of sacrifice (day 39 after inoculation) and fixed in 10% neutral buffered formalin and processed for proliferating cell nuclear antigen (PCNA). Representative images of PCNA staining are shown in the upper panel (400x). Quantification of the positive rate is shown in the lower panel. *n* = 20 in each group. ****p < 0.0001. **(C)** Representative images of CD31 staining in tumor xenografts are shown in the upper panel (400x). Microvessel density (MVD) counts are shown in the lower panel. *n* = 20 in each group. ****p < 0.0001. **(D)** Representative images of terminal deoxynucleotidyl transferase dUTP nick end labeling (TUNEL) are shown in the upper panel (400x). TUNEL counts are shown in the lower panel. *n* = 20 in each group. *p* > 0.05. Data are presented as mean ± SD. HPF: high power field.

To clarify the therapeutic effect of BIX01294 on tumor growth in human cervical cancer cells, SiHa cell line xenograft tumors were used as a cervical cancer model. After xenograft tumors (each about 64 mm^3^) formed, vehicle (normal saline) or different doses of BIX01294 were used to treat the mice twice a week. After inoculations, each mouse produced one xenograft tumor. The tumor growth curve revealed that administering 10 mg/kg of BIX01294 significantly reduced SiHa cell line xenograft tumor growth (Figure 10A). Meanwhile, we used sections of xenograft tumors in the following determinations: *in vivo* cell proliferation status by proliferating cell nuclear antigen (PCNA) immunohistochemical staining (Figure 10B), microvessel density (MVD) by CD31 staining (Figure 10C), and tumor cell apoptosis by terminal deoxynucleotidyl transferase dUTP nick end labeling (TUNEL) assay (Figure 10D). A total of 30 xenograft tumors were used for these studies. Quantitative results revealed that BIX01294 significantly reduced cervical cancer cell proliferation and tumor angiogenesis but did not significantly influence tumor cell apoptosis *in vivo*. Overall, Figure 10 shows that G9a may promote tumor growth in mouse model.

### G9a and the clinical survival rate

**Figure 11:**
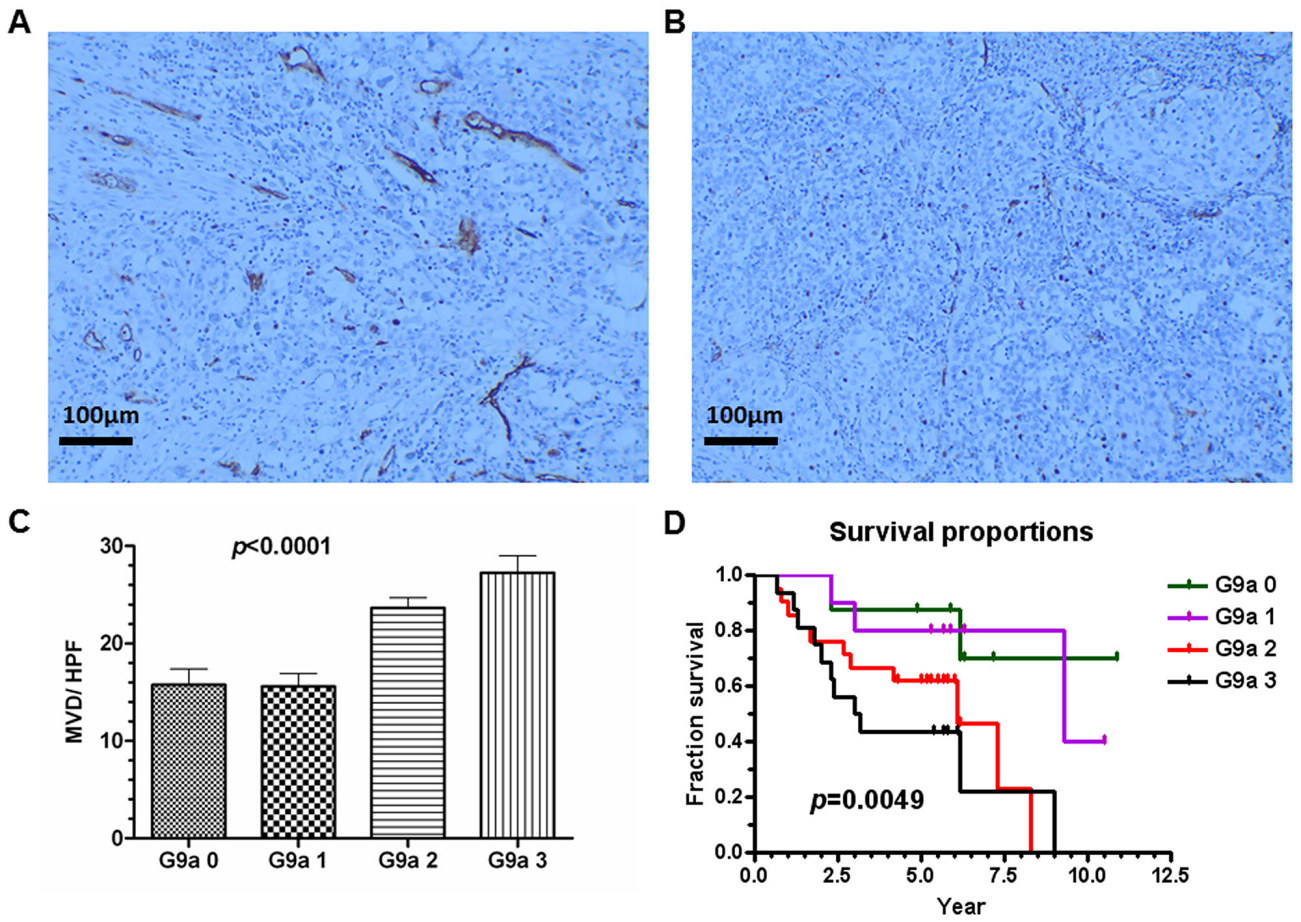
G9a expression correlates with microvessel density (MVD) and with poor clinical survival rate G9a expression in human cervical cancer tissue was scored on a scale ranging from negative (grade 0) to positive (grades 1 to 3). MVD was counted by CD31 staining under HPF (100x). **(A)** Example of high MVD (26/HPF). **(B)** Example of low MVD (6/HPF). **(C)** The correlation of MVD and G9a levels in human cancer tissue. Total *n* = 275, *p* < 0.0001 (one-way ANOVA). **(D)** Survival proportions and G9a expression patterns were calculated. Total *n* = 55, *p* = 0.0049 (Logrank test for trend). HPF: high power field.

We are interested in discovering a correlation between G9a expression and clinical outcome. In our hospital, between January 1998 and December 2003 there were a total of 231 stage I or II cervical cancer patients who underwent radical surgery and had regular follow-ups. By December 2013, 199 (86.1%) were survivors and 32 (13.9%) had already died of the disease. Surgical tissue specimens from the deceased patients and matched survivors were used to examine G9a expression. Each deceased patient was matched with a survivor for tumor stage, age, and nearest operation date; of the 64 matched cases, 55 archived formalin-fixed cervical carcinomas (deceased, 27; surviving, 28) were available for study. Of these 55 cases, 8 (14.5%) were G9a expression negative (grade 0); 10 (18.2%), grade 1 positive; 21 (38.2%), grade 2 positive; and 16 (29.1%), grade 3 positive (Supplementary Table 3). MVD in the human tumors was assessed by CD31 staining (Figure 11A & 11B). The expression of CD31 in cervical cancer showed a significant correlation with G9a (Figure 11C). Survival analysis showed that the higher the exhibited G9a expression, the poorer the survival time (Figure 11D). Overall, Figure 11 indicates that the expression levels of G9a clinically correlate with angiogenesis, tumor progression and patient survival.

## DISCUSSION

Immunohistochemical studies of G9a have been reported for other cancers. For prostate cancer, histone acetylation and dimethylation in histones H3 and H4 are predictors of outcome independent of tumor stage [33]. In esophageal squamous cell carcinomas, G9a expression is a clinicopathological parameter [34]. In our study, we found that, in contrast with other cancer precursors, G9a and H3K9me2 were highly expressed in cervical cancer cells. A higher expression of G9a in tumor tissue correlated with a lower patient survival rate. To the best of our knowledge, G9a’s angiogenic activity is not well known and this paper is the first study that investigates how G9a functions in cervical cancer.

For cell proliferation in fibroblast-like cell lines (COS-7 cells), G9a was found to provide a mechanism for coordinating DNA and H3K9 methylation during cell division [35]. In fetal pulmonary arterial smooth muscle cells, BIX01294 treatment was reported as blocking cell proliferation [36]. In mouse models of acute myeloid leukemia, G9a was highlighted as possessing clinical potential as an inhibitor of proliferation. Activation of serine-glycine synthesis by G9a was also found to be essential to sustaining cancer cell proliferation [25]. Down-regulation of G9a was said to trigger a DNA damage response and thus inhibit colorectal cancer cell proliferation [37].

Modification of H3K9 has been suggested as promoting cancer invasion and metastasis in the human breast cancer cell line MDA-MB-231 [38], the lung adenocarcinoma cancer cell lines CL1-0 and CL1-5 [21], the pancreatic cancer cell line PANC-1 [39], and the ovarian cancer cell line SKOV-3 [40]. In germ cell tumors, G9a was reported as being linked to hypoxia-regulated epigenetic changes and to the inhibition of G9A, which may have resulted in dysregulation of angiogenesis [41]. In oral squamous cell carcinoma, inhibition of G9a is said to induce autophagy and apoptosis [42]. Surprisingly, when we checked cell apoptosis by flow cytometry, cell cycle analysis, FITC annexin V and PI labeling, TUNEL assay, active caspase-3 measurement, and apoptosis cell array we found that, overall, BIX01294 treatment did not have any effect on apoptosis. Whether G9a possesses a different effect on apoptosis for various cancers needs further investigation.

H3K9 histone methyltransferase G9a may promote tumor invasion and metastasis [21, 24]. In head and neck squamous cell cancers, G9a was said to be essential for metastasis *via* E-cadherin repression [24]. Previously, we found that interleukin-8 is an important angiogenic factor related to the activation of the lysophosphatidic acid receptors LPA2 and LPA3 [29]. Recently, interleukin-8 was shown to be a downstream effector of G9a [39]. Also, inhibition of EHMT2/G9a may promote Beclin-1 transcription through activation of NF-κB [26]. A distinct mechanism study revealed interplay between DNA methylation and histone modification and a dual recognition of H3K9me2 marks by BAH and chromodomain [43]. Here, we’ve shown that G9a may promote angiogenesis through multiple factors. Overall, as is shown in both the *in vitro* and *in vivo* assays, these angiogenic factors may promote angiogenesis. We’ve used the interleukin-8 promoter-reporter assay to show that G9a promotes angiogenic gene expression by transcriptional regulation. Interleukin-8 is a target gene regulated by G9a in cervical cancer cells. However, further research is necessary in order to understand how G9a’s inhibition leads to the downregulation of all 14 of these angiogenic factors.

DNA hypermethylation may enhance anticancer drug resistance [44]. Targeting histone lysine methylation pathways has been suggested as being a relevant, emerging cancer therapeutic strategy [45]. Previously, it has been reported that use of UNC0638, another G9a inhibitor, markedly reduced the clonogenicity of a breast cancer cell line (MCF7 cells) [46]. G9a inhibition impaired cancer cell growth for liver cancer, too [47]. In our study, we have shown that BIX01294 may down regulate G9a, reduce clonogenicity, repress angiogenesis, and retard cancer tumor growth while not affecting cell apoptosis. Because G9a has a broad-spectrum involvement in tumorigenesis through cancer cell proliferation, tumor angiogenesis, and tumor invasion/metastasis, BIX01294 may have potential as an agent for acting on specific molecular targets.

## MATERIALS AND METHODS

### Tissue array and cervical cancer tissue for G9a staining

For G9a immunohistochemical staining, we used formalin-fixed arrays of normal, benign, and malignant cervical tissue from Pantomics, Inc. (San Francisco, CA) (CXC961, CXC962, CXC1021, CIN481, CIN482, and CIN483, of which there were no overlapping cases) and paraffin-embedded tissue blocks of cervical carcinoma removed surgically by radical hysterectomy. Of the six arrays, three (CXC961, CXC962, and CXC1021) had previously been used to study BCL10 [48]. Normal cervical specimens obtained surgically from benign uterine corpus or ovarian disease were also used.

Tissue sections were deparaffinized in xylene and rehydrated using graded concentrations of alcohol to distilled water. After antigen retrieval by heat treatment in a 0.1 M citrate buffer at pH 6.0, endogenous peroxidase activity was blocked using a 3% H_2_O_2_ solution. Slides were then incubated for 30 min in 2.5% normal donkey serum or goat serum. Afterward, the slides were incubated overnight at 4°C with monoclonal antihuman G9a (R&D Systems, Minneapolis, MN), followed by incubation with secondary antibodies according to the manufacturer’s instructions. Finally, antibody binding was detected using the avidin-biotin-peroxidase method. Reaction products were developed using 3’, 5’-diaminobenzidine (Dako, Glostrup, Denmark) as a substrate for peroxidase. Sections were counterstained with Mayer’s hematoxylin. All washes were performed using a phosphate-buffered saline solution (pH 7.4). Nuclear staining was considered as positive for G9a staining. The expression level of G9A was classified as either negative (grade 0) or positive (grades 1 to 3). A positive staining less than or equal to 20% of cells was grade 1; between 20-50% of the cells, grade 2; and more than 50% of cells, grade 3.

### Cell culture

All cervical cancer cell lines were obtained from the American Type Culture Collection (Rockville, MD). The following cell lines were maintained in DMEM (Life Technologies, NY): SiHa (squamous carcinoma, cervix, human) and HeLa (epitheloid carcinoma, cervix, human). Concurrently, CaSki (epidermoid carcinoma, cervix, human) was maintained in a RPMI 1640 medium (Life Technologies, NY). All culture media were supplemented with 10% (vol/vol) fetal bovine serum (FBS), penicillin, and streptomycin in a humidified atmosphere of 95% air and 5% CO_2_ at 37°C. Human cervical epithelial cells were obtained from Cell Application and were cultured in an epithelial cell culture medium (Cell Application). Human umbilical vein endothelial cells (HUVECs) were also obtained from the American Type Culture Collection (Rockville, MD). HUVECs were cultured in a M199 medium supplemented with 20% FBS, endothelial cell growth supplement (Intracel, Rockville, MD), heparin, L-glutamine, penicillin, and streptomycin in a humidified atmosphere of 95% air and 5% CO_2_ at 37°C. The following experiments were performed using HUVECs at no more than five passages. The chemical inhibitor, BIX01294, was purchased from Sigma (St Louis, MO).

### Antibodies

Antibodies used for immuno-blotting were SC-7943 (for cIAP-1), SC-1060 (for HSP70), SC-8789 (for XIAP) and SC-55778 (for GADPH). All were purchased from Santa Cruz Biotechnology (Santa Cruz, CA). Neutralizing antibodies for CXCL16, interleukin-8, angiogenin and TIMP-1 were also purchased from Santa Cruz Biotechnology. The G9a antibody (PP-A8620A-00) was purchased from R&D Systems. The H3K9me2 (D85B4) and histone H3 antibodies (#9715) were purchased from Cell Signaling Technology (Beverly, MA).

### Western blotting

For total cell lysate extraction, cervical cancer cells were lysed in a lysis buffer (Thermo Fisher Scientific, Waltham, MA). Cell lysates were then centrifuged at 30,000 x g for 25 minutes at 4°C. Nuclear proteins were extracted using NE-PER Nuclear and Cytoplasmic Extraction Reagents (Thermo Scientific Pierce, Thermo Fisher Scientific, Waltham, MA, US). The protein concentration was measured with a Bio-Rad protein assay. A 10 to 50 μg protein sample was separated using a 10%∼15% sodium dodecyl sulfate polyacrylamide gel electrophoresis and then transferred onto a polyvinylidene difluoride membrane. The membranes were blocked with 5% fat-free milk for 30 minutes. They were then immunoblotted with various primary antibodies (described in Antibodies) (1:1000 dilution) for 1 hr at room temperature. Bound antibodies on the membrane were detected using appropriate peroxidase-coupled secondary antibodies for 30 minutes. The signal development was achieved by addition of the Immobilon Western Chemiluminescent HRP Substrate (WBKLS0500, Millipore). A digital imaging system (Bio Pioneer Tech Inc.) was used to detect and digitally capture the signals.

### MTT assay

Cells were plated onto 96-well microplates at a density of 5 x 10^3^ cells/well for a cell viability and proliferation assay. The cells were cultured at 37°C for the indicated time; afterward, 30 μl of MTT solution (5 mg/ml) was added into each well and then incubated for 4 hrs in darkness. The formazan grain was then dissolved in DMSO, and the absorbance at 570 nm was read using an ELISA plate reader.

### Flow cytometry for cell cycle analysis and detection of apoptosis

A FACS scanner and Cell Quest software (Becton Dickinson Immunocytometry Systems, San Jose, CA) were used to determine cell cycle and the status of apoptosis by quantifying Annexin V/PI staining. Staining was quantified according to manufacturer’s instructions from the Alexa Fluor® 488 Annexin V/Dead Cell Apoptosis Kit (Thermo Fisher Scientific Inc.).

### Active caspase-3 measurement

The active caspase-3 levels of the cell lysate were determined using the manufacturer’s instructions from a commercially available Human Active Caspase-3 Immunoassay kit (KM300) purchased from R&D Systems. Each measurement was repeated in triplicate. We used staurosporine-treated cancer cells as a positive control for apoptosis assays (because staurosporine may induce apoptosis) [49].

### Preparation of conditioned medium

Cultures of SiHa cells (2 × 10^6^/10-mm dish) were rinsed twice with PBS and cultured in 5 mL of serum-free DMEM. The SiHa cells were then treated with 5 μM of BIX01294 for 4 hrs; after washing out the medium, the cells were incubated in fresh culture medium for 24 hrs to collect conditioned medium. Collected conditioned medium was then clarified by centrifuge (4°C, 10 000 rpm, 5 min) to remove cell debris. A final solution of 25 mM HEPES buffer (pH 7.4), 1 mg/mL leupeptin, 1 mM phenylmethylsulfonyl fluoride, 1 mM EDTA, 0.02% NaN_3_, and 0.1% BSA (Sigma) was added. The conditioned medium was then frozen and stored at −70°C until used.

### Protein array analysis

The conditioned medium from BIX01294- or vehicle-treated SiHa cells was incubated with angiogenesis antibodies for angiogenesis array (R&D systems). The cell lysate of BIX01294- or vehicle-treated SiHa cells was incubated with human apoptosis antibodies for apoptosis array (R&D systems). Membranes were washed several times with a specific buffer, treated with biotin-conjugated antibodies for 1 to 2 hrs, washed again, and then treated with diluted horseradish peroxidase–conjugated streptavidin for 2 hrs and with specific detection buffer at room temperature for 1 min. A chemiluminescent imaging system was used to detect array signals.

### siRNA transfection

Optimal siRNAs were purchased from Santa Cruz Biotechnology, Inc. (Santa Cruz, CA). The targeted siRNA for G9a and control siRNA were sc-43777 and sc-37007, respectively. Cervical cancer cell lines were transfected with siRNA using the siRNA Transfection Protocol (Santa Cruz Biotechnology).

### Transient transfection of the G9a over-expression vector

Human G9a expression vector pWPXL-G9a-Myc-DDK was constructed and provided by Dr. Min-Wei Chen. To construct pWPXL-G9a-Myc-DDK, the sequence encoding G9a-Myc-DDK was excised from pCMV6-EHMT2-Myc-DDK (Cat. No.: RC219200, Origen) and inserted into pWPXL downstream of the EF1α promoter. HeLa, SiHa and CaSki cells were transient transfected with 5 μg of pWPXL-G9a-Myc-DDK or empty vector using the TransFastTM transfection reagent (Promega Corporation, Madison, WI) according to manufacturer’s instructions.

### Real-time quantitative RT-PCR

Total RNA was isolated from cultured cell lines using a RNAzol B reagent (Biotecx Laboratories, Houston, TX) according to the manufacturer’s instructions, and then cDNA was prepared from 2 μg of the total RNA with random hexamer primers according to the cDNA synthesis ImProm-II protocol (Promega). The specific oligonucleotide primer pairs for human CXCL16 were forward 5′-GAG CTC ACT CGT CCC AAT GAA-3′, reverse 5′-TCA GGC CCA ACT GCC AGA-3′; for human beta-actin, forward 5′-GCC AAC CGC GAG AAG ATG A-3′, reverse 5′-CAT CAC GAT GCC AGT GGT A-3′; for interleukin-8, forward 5′-CCA GGA AGA AAC CAC CGG-A-3′, reverse 5′-GAA ATC AGG GCT GCC AAG-3′; for human angiogenin, forward 5′-CCT GGG CGT TTT GTT GTT GG-3′, reverse, 5′-TGT GGC TCG GTA CTG GCA TG-3′; and for human G9a, forward 5′-CCG GCG CAA GGC CAA GAA GA-3′, reverse 5′-CGG TGG GCC ACA CGG AAG TC-3′. The amplification program consisted of one cycle of an initial incubation at 61°C for 20 min, followed by 50 cycles of denaturation at 95°C for 10 sec, annealing at 55–57°C for 10 sec, and then extension at 72°C for 10 sec. The amount of indicated mRNA was normalized by that of glyceraldehyde-3-phosphate dehydrogenase (GAPDH) mRNA and is presented in arbitrary units, with 1 U corresponding to the value in cells treated with a vehicle control.

### Enzyme immunoassay (EIA)

Using commercially available kits, we used EIA to determine the CXCL16, interleukin-8 and angiogenin levels of the cell culture supernatant. Product numbers DCX160 (for CXCL16), D8000C (for interleukin-8), and DAN00 (for angiogenin) were purchased from R&D Systems. Each measurement was repeated in triplicate.

### HUVECs monolayer permeability assay

HUVECs were cultured in Transwell chambers (0.4 μm pore polycarbonate filters; Costar, Cambridge, Miss.). After reaching confluence, the medium was replaced with the previously-collected conditioned medium (0.3 ml in the upper chamber and 1 ml in the lower chamber). Horseradish peroxidase molecules (type VI-A, 44 kDa; Sigma-Aldrich) at a concentration of 0.126 μM were then added to the upper compartment. After incubating for 1 hr, the medium in the lower compartment was assayed for enzymatic activity using a photometric guaiacol substrate assay (Sigma-Aldrich).

### HUVECs migration assay

HUVECs were seeded into the inserts of transwell (8 μm pore) dishes and were treated with the previously-collected conditioned medium. The cells were allowed to migrate toward the lower chamber for 6 hrs. Non-migrated cells were removed from the upper surface of the membrane, while cells that had migrated to the lower surface were fixed and stained with crystal violet. Migration cells were digitally photographed and counted per high power field (HPF).

### HUVECs proliferation assay

HUVECs were plated onto six-well cell culture plates at 1 x 10^5^ cells/well in 2 ml of culture medium with the previously-collected conditioned medium. After 72 hrs at 37°C, the cells were harvested by suspending them in a 0.025% trypsin/0.02% EDTA solution. Cell counts were performed in triplicate using a hemocytometer; trypan blue exclusion was used to identify viable cells.

### HUVECs endothelial cell tube formation assay

To evaluate polygonal vascular tubular formations, HUVECs were plated (2 x 10^5^ cells/ml) onto a layer of Matrigel (0.24 mg/cm^2^) with the conditioned medium that was collected earlier (described above). After 6 hrs, three replicate fields of triplicate wells were digitally photographed and counted.

### *In vitro* wound healing migration assay

Confluent SiHa cells were grown in 6-well plates at a density of 2 × 10^6^/mL and pretreated with 5 μM of BIX01294 for 24 hrs prior to an *in vitro* wound healing migration assay. A small linear scratch was created in the confluent monolayer by gentle scraping with a sterile cell scraper. Cells were rinsed extensively with medium to remove cellular debris and then incubated with DMEM supplemented with 10% FBS. Six hrs later, images of the migrated cells were digitally photographed. Distances cells had migrated into the denuded area were determined.

### Transwell invasion assay

SiHa cells were seeded into inserts of Matrigel (Corning Matrigel matrix) coated transwell (8 μm pore) dishes and were treated with vehicle or BIX01294. These cells were allowed to invade/migrate for 16 hrs toward the lower chamber. Non-migrated cells were removed from the upper surface of the membrane, while cells that had migrated to the lower surface were fixed and stained with crystal violet. Migrated cells were digitally photographed and counted per HPF.

### *In vivo* **CAM** assay

For experiments dealing with intravasation, the SiHa cells that had been treated with BIX01294 or vehicle were detached from the culture dish with 2 mM EDTA in PBS, counted, and then suspended again in PBS; 50 μl (1 x 10^6^ cells) of these SiHa cells were then inoculated onto each CAM from a 9-day-old chick embryo for which an artificial air sac had been created (upper CAM, *n* = 5). After incubating for 48 hrs, the eggs were frozen at −20°C. The lower half of the CAM (lower CAM) was then removed. The lower CAM was used for DNA extraction employing a DNA Isolation Kit (Promega).

For the detection of repeat Alu sequences in the CAM assay, 50 ng of DNA was assayed using primer sequences sense 5′; ACG CCT GTA ATC CCA GCA CTT 3′ and antisense 5′; TCG CCC AGG CTG GAG TGC A 3′. A specific primer for human Alu sequences was positioned in the most conserved areas of the Alu sequence. PCR conditions used were 95 °C for 120 sec, 40 cycles of 95°C for 30 sec, 62°C for 30 sec, and then finally 72°C for 45 s with a 72°C hold for 300 sec, which produced a band of 224 bp. The chGAPDH was used as an internal control; the primers were sense 5′; GAG GAA AGG TCG CCT GGT GGA TCG-3′ and antisense 5′; TCA GCT CAG GGA TGA CTT TC 3′. PCR conditions used were 95°C for 120 sec, 20 cycles of 95 °C for 30 sec, 58°C for 30 sec, and then finally 72°C for 45 sec with a 72°C hold for 300 sec, which produced a band of 194 bp (from the region 266–460 (NM_204305). PCR products were electrophoresed on a 1.8% agarose gel at 100 V and visualized using ethidium bromide. Images of PCR products were then obtained and analyzed by Image J.

### Animal studies

The protocol for xenograft experiments was approved by the National Taiwan University College of Medicine and the National Taiwan University College of Public Health Institutional Animal Care and Use Committee on January 3, 2013 (Affidavit of Approval of Animal Use Protocol No. 20120383). Thirty female, 6-week-old, severe combined immunodeficient (SCID) mice were used for a cervical cancer ectopic xenograft model (*n* = 10 per group). In this ectopic xenograft cervical cancer tumor model, cervical cancer cells (1 x 10^6^) were injected subcutaneously into the right hind limb of SCID mice. By the end of the experiments, the vehicle (control) group tumors attained an average volume of 500 mm^3^. The therapeutic effect of BIX01294 on this ectopic xenograft model was measured using tumor growth curves. Tumor volume was measured twice a week, and tumor volumes were calculated using a standard formula: width^2^ × length /2. The therapeutic effect of BIX01294 was compared with the vehicle treatment.

### Immunohistochemical staining

Slides were re-hydrated in PBS for 15 min, and the endogenous peroxidase was then inhibited using 3% H_2_O_2_/methanol for 10 min at room temperature. For blocking, 5% non-fat milk/PBS was used for 30 min at room temperature. Slides were incubated with either anti-human PCNA (sc-56, Santa Cruz Biotechnology), CD31 (sc-1506, Santa Cruz Biotechnology) antibodies or a TUNEL reagent (R&D Systems) for 16 hrs at 4 °C. The peroxidase-conjugated secondary antibody was incubated for 1 hr at room temperature and developed by immersing slides in 0.06% 3,3’-diaminobenzidine tetrahydrochloride, followed by counterstaining with Gill’s hematoxylin.

### PCNA, MVD and TUNEL assessment

Tumors from vehicle and BIX01294 (10 mg/kg) treatment groups were harvested at the time of sacrifice (day 39 after inoculation), fixed in 10% neutral buffered formalin, and then processed for PCNA, MVD and TUNEL assessment. One tumor was obtained from each mouse (10 tumors in each group; 20 tumors in total for comparison). Each PCNA, MVD or TUNEL measurement was made using one tissue section from each tumor. Immunohistochemical reactions were first observed under low-power field (LPF, 40x), and two LPFs were chosen for counting under HPF (400x; 0.152 mm^2^; 0.44-mm diameter). Positive stainings of PCNA, CD31 and TUNEL were counted in 5 representative HPFs. The mean value of the HPFs was used as the final count of that LPF. As a result, each group had 20 countings of either PCNA, CD31 or TUNEL that was used for statistical evaluation. Single immunoreactive cancer cells with signs of PCNA or TUNEL (dark brown) in the nucleus were counted. Single immunoreactive endothelial cells, or endothelial cell clusters separate from other microvessels, were counted as individual microvessels. Endothelial staining of large vessels with tunica media and nonspecific staining of nonendothelial structures were disregarded in microvessel counts. The mean visual CD31 staining for MVD was calculated.

### Survival analysis

Between 1998 and 2003, 231 patients with stage I or II cervical carcinoma underwent a radical hysterectomy at our hospital. By December 31, 2013, they had been completing follow-ups for at least 10 years and thus became candidates for our study. Archived surgical specimens from each deceased patient and a matched survivor were used for immunohistochemical staining of G9a and analyzed for overall survival. This study was approved by our Institutional Research Ethics Committee (Approval for Clinical Research 201211033RIB, issued by Research Ethics Committee B of the National Taiwan University Hospital on January 2, 2013).

### Statistical analysis

Data are presented as mean ± standard deviation (SD). The Student-*t* test, ANOVA, chi-square test and chi-square test for trend are used for statistical analysis of continuous or categorical variables. Survival curve analysis is made by the Kaplan-Meier method. Two-tailed *p*-values less than 0.05 are considered significant.

## SUPPLEMENTARY MATERIALS FIGURES AND TABLES


