# Supplementary material for: Methyltransferase G9a promotes cervical cancer angiogenesis and decreases patient survival
Source: Oncotarget. 2017 Jul 7;8(37):62081–98. doi: 10.18632/oncotarget.19060 (PMC5617488; doi:10.18632/oncotarget.19060)
Supplement: Supplementary file 1 [file oncotarget-08-62081-s001.pdf]

Methyltransferase G9a promotes cervical cancer angiogenesis and decreases patient survival

SUPPLEMENTARY MATERIALS

H3K9me2 immunohistochemical staining of human cervical cancer tissue

A cervical cancer tissue array section (CXC1021, US Biomax) containing both cervical cancer and normal tissue was deparaffinized in xylene and rehydrated using graded concentrations of alcohol to distilled water. After antigen retrieval by heat treatment in a 0.1 M citrate buffer at pH 6.0, endogenous peroxidase activity was blocked using a 3% H2O2 solution. Slides were then incubated for 30 minutes in 2.5% normal donkey serum or goat serum. Afterward, the slides were incubated overnight at 4°C with an antibody against H3K9me2 (D85B4), followed by incubation with secondary antibodies according to the manufacturer's instructions. Finally, antibody binding was detected using the avidin-biotin-peroxidase method. Reaction products were developed using 3', 5'-diaminobenzidine (Dako, Glostrup, Denmark) as a substrate for peroxidase. Sections were counterstained with Mayer's hematoxylin. All washes were performed using a phosphate-buffered saline solution (pH 7.4). Nuclear staining was considered as positive for H3K9me2 staining.

Evaluations of the efficiency of G9a siRNA

G9a siRNA efficiency was confirmed by detecting G9a mRNA expression *via* q-RTPCR (as described) using Real-time quantitative RT-PCR. G9a siRNA efficiency for G9a protein expression was confirmed using western blot as described above.

Human interleukin-8 (IL-8) promoter-reporter assay

To evaluate the effect G9a has on IL-8 promoter activity, cervical cancer cells were treated with G9a siRNA or control siRNA for 24 hrs. These cells were then transfected with human IL-8-1.4 Kb promoter-driven luciferase plasmids (BD Bioscience) using the Transfast transfection reagent (Promega). At 24 hrs after transfection, the luciferase activities were determined by Luminescence Reader (Beckman DTX880).

MVD assessment in cervical cancer tissue

Cervical cancer tissue sections from survival proportions and G9a expression pattern groups were further used for CD31 staining for micro-vessel density assessment. Slides were processed for immunohistochemical staining. The slides were incubated overnight at 4°C with antihuman CD31 (Santa Cruz Biotechnology); immunohistochemical reactions for CD31 were observed at LPF (40x) and positive stainings of CD31 were counted in 5 representative HPFs (400x; 0.152 mm^2; 0.44-mm diameter) for each tumor section. Single immunoreactive endothelial cells, or endothelial cell clusters separate from other microvessels, were counted as individual microvessels. Endothelial staining of large vessels with tunica media and nonspecific staining of nonendothelial structures were disregarded in microvessel counts. The mean visual CD31 staining for MVD was calculated.

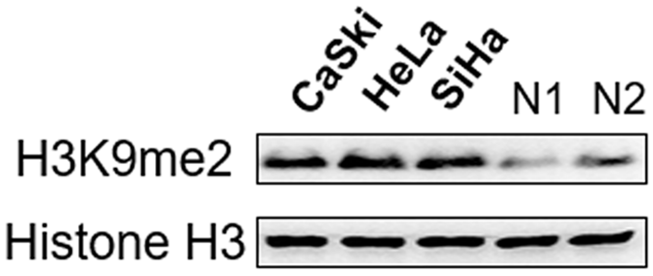

Supplementary Figure 1: H3K9me2 expression in cervical cancer cells. Western blot analysis of nuclear lysates from cancer and normal human cervical epithelial cells (N1 and N2; 2 different lots) for H3K9me2 expression. Detection by western blot. Histone H3 was used as a loading control.

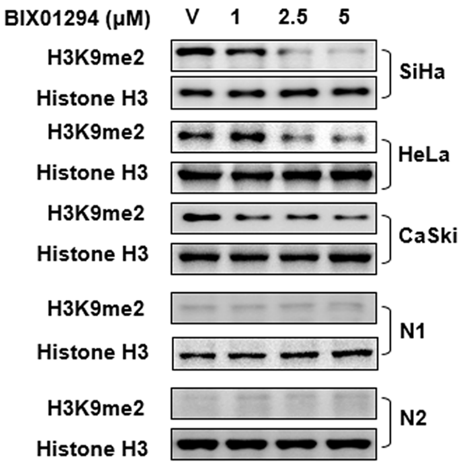

Supplementary Figure 2: Effect of G9a chemical inhibitor BIX01294 on the methylation of H3K9 in cervical cancer cells. SiHa, HeLa and CaSki cells and two normal human cervical epithelial cells (N1 and N2) were treated with indicated dose of BIX01294 for 24 hrs. Protein levels for HK9me2 and histone H3 were detected by western blot. Histone H3 was used as a loading control.

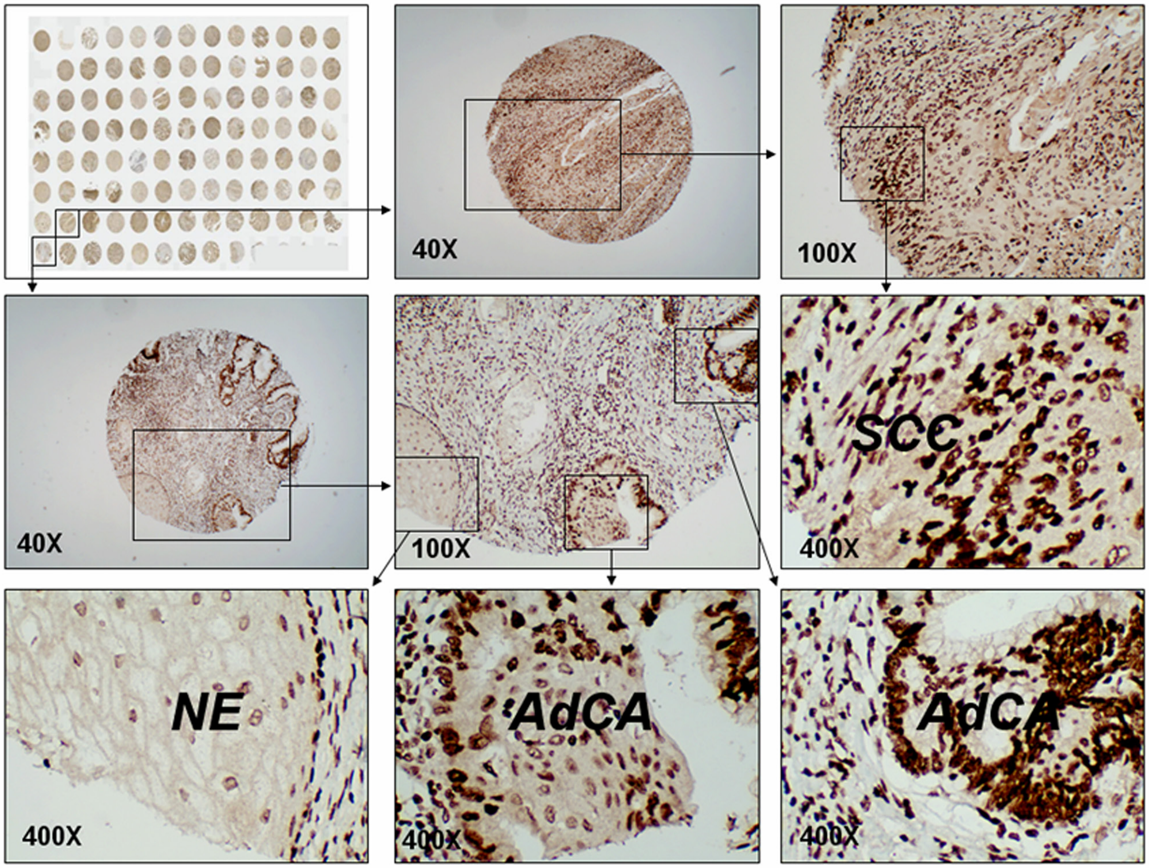

Supplementary Figure 3: Immunohistochemical staining of H3K9me2 in cervical cancer. To confirm differences in H3K9me2 expression for normal and cancer cells, a cervical cancer tissue array (CXC1021, US Biomax) containing both cervical cancer and normal tissue was stained using the H3K9me2 antibody. In contrast to normal cervical epithelium (***NE***) where H3K9me2 presented only a weak staining, a strong and condensed nuclear brown staining was found for adenocarcinoma (***AdCA***) and squamous cell carcinoma (***SCC***) cancer cells.

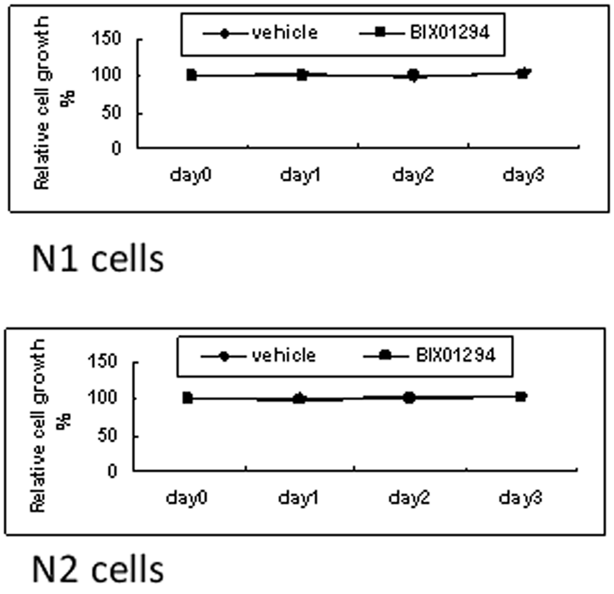

Supplementary Figure 4: Effect of BIX01294 on the cell growth of normal human cervical epithelial cells. Normal human cervical epithelial cells (N1 and N2) were treated with 5 μM of BIX01294 at different times; viable cells were determined by MTT assay. The relative cell growth rates from day 0 were calculated. $n = 3$, $p > 0.05$.

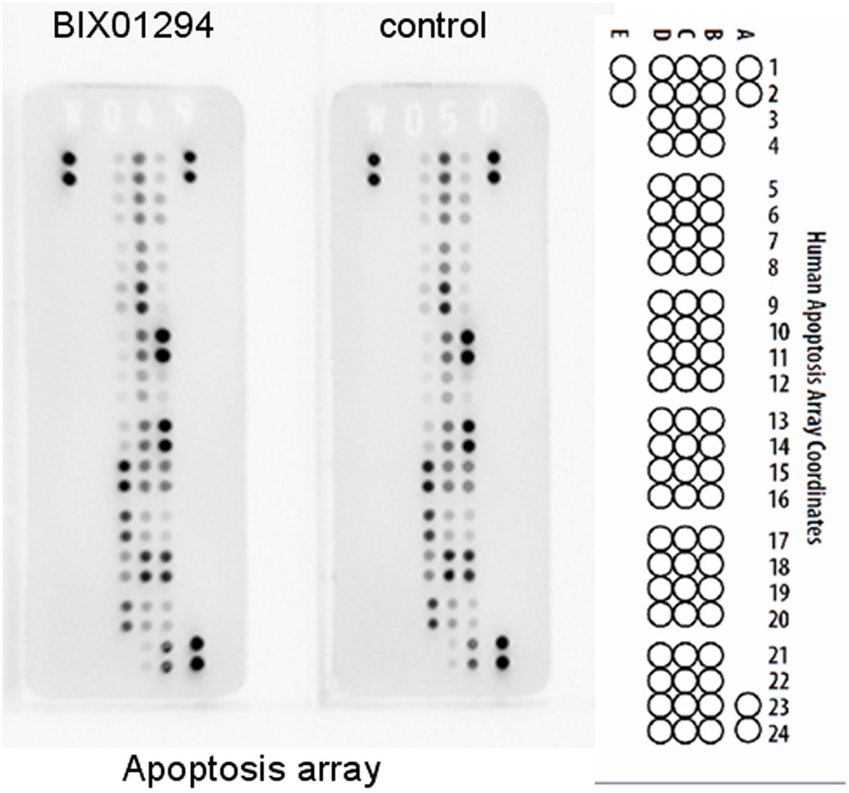

Supplementary Figure 5: Effect of BIX01294 on apoptosis-related protein expression in SiHa cells. SiHa cells were treated with BIX01294 for 4 hrs. Total cell lysate was used for apoptosis related protein expression pattern analysis. Representative figures (left panel) and coordinates (right panel) are shown. (BIX01294, $n = 3$; control, $n = 3$).

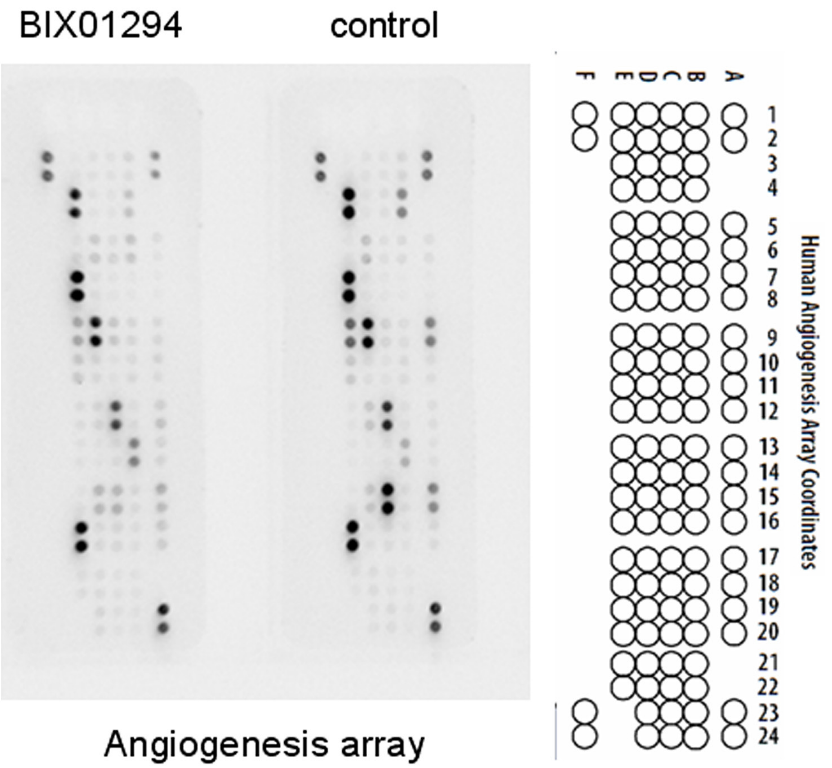

Supplementary Figure 6: Effect of BIX01294 on angiogenesis-related protein expression. SiHa cells were treated with BIX01294 for 4 hrs; after washing out the medium, cells were incubated in fresh culture medium for 24 hrs to collect conditioned medium. Conditioned medium was used for angiogenic factor analysis. Representative figures (left panel) and coordinates (right panel) are shown. (BIX01294, $n = 3$; control, $n = 3$).

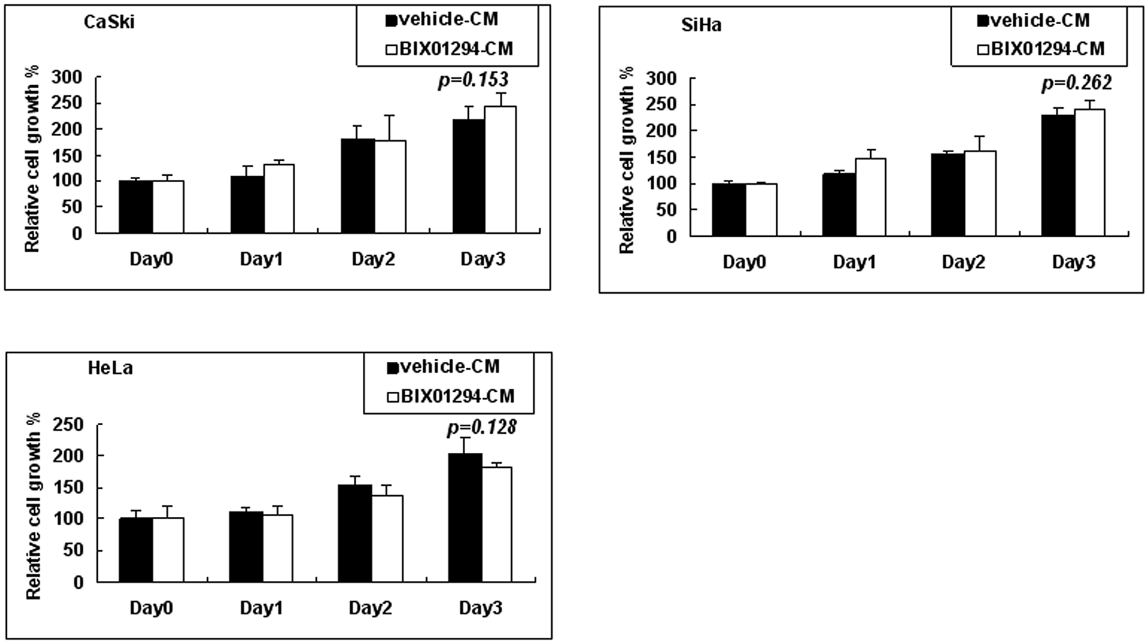

Supplementary Figure 7: Effect of angiogenic factors on proliferation of cervical cancer cells. Cervical cancer cells (SiHa, HeLa and CaSki) were treated with 5 μM of BIX01294 or vehicle; conditioned medium was used to culture their own cancer cell lines. At different times, viable cells were determined by MTT assay. The relative cell growth rates from day 0 were calculated. $n = 3$, all $p > 0.05$ (vehicle-CM *vs.* BIX01294-CM). vehicle-CM: conditioned medium from vehicle-treated cells. BIX01294-CM: conditioned medium from BIX01294-treated cells.

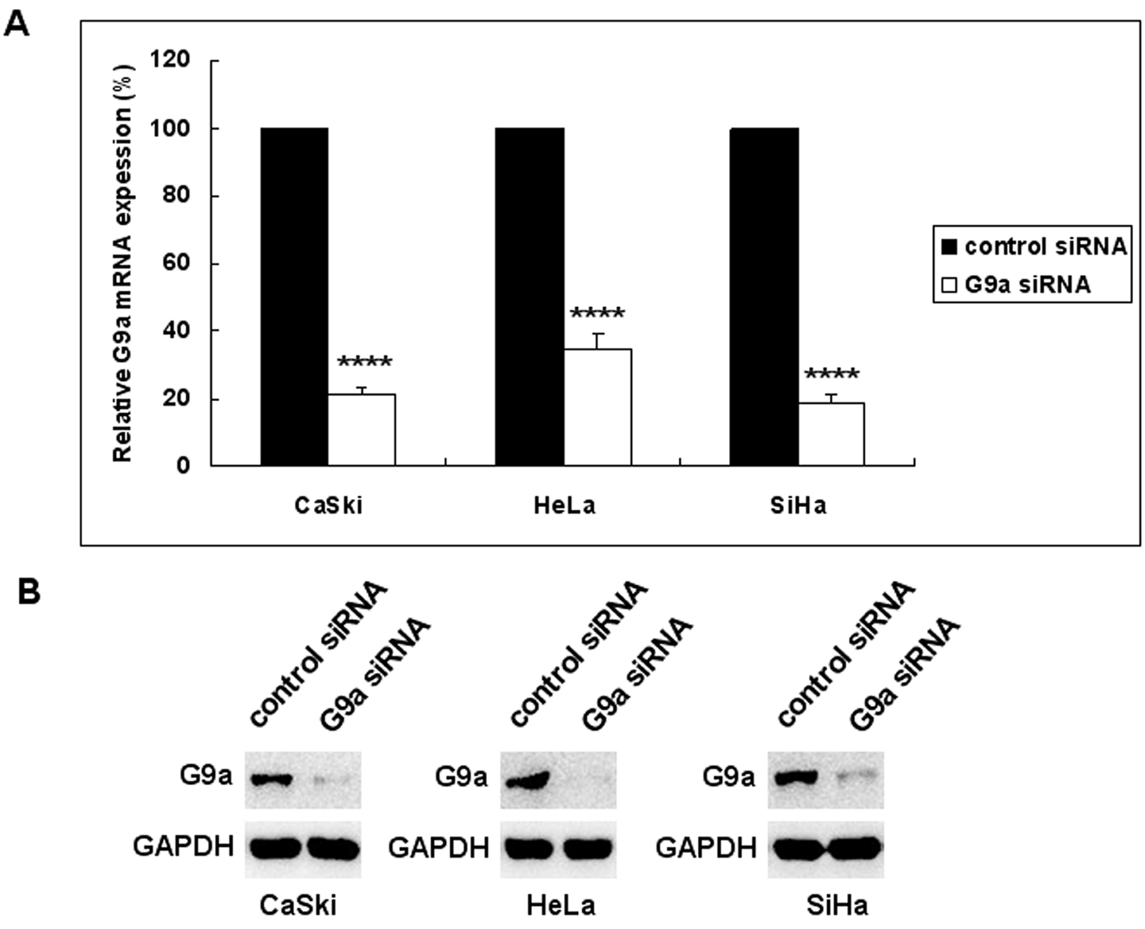

Supplementary Figure 8: Efficiency of G9a gene knockdown. G9a siRNA efficiently decreased the relative G9a mRNA expression **(A)** and G9a protein levels **(B)**. Control siRNA: transfection with control siRNA sc-37007. G9a siRNA: transfection with G9a siRNA sc-43777.

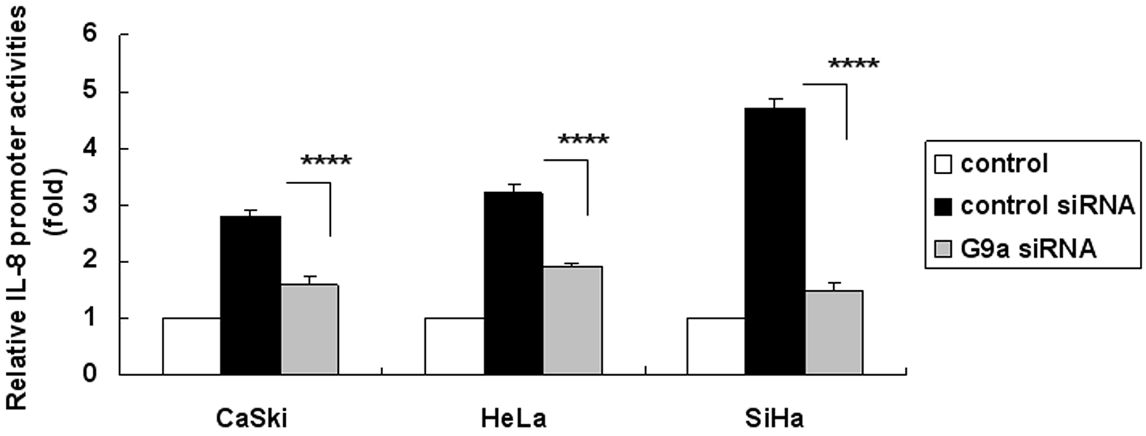

Supplementary Figure 9: Interleukin-8 promoter assay. We used the interleukin-8 promoter assay to clarify the inhibition effect of G9a siRNA on the transcriptional regulation of interleukin-8. Interleukin-8 promoter activity was represented by fold of luciferase activity. $n = 6$, ****$p < 0.0001$. Control: without transfection. Control siRNA: transfection with control siRNA sc-37007. G9a siRNA: transfection with G9a siRNA SC43777. IL-8: interleukin-8.

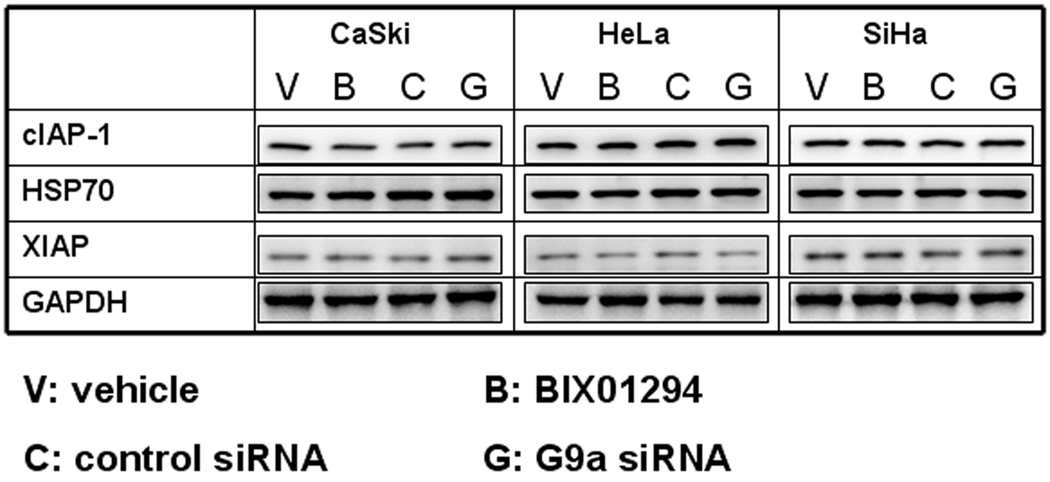

Supplementary Figure 10: Confirmation of the effect of G9a chemical inhibitor BIX01294 on apoptosis-related protein expression. Cervical cancer cells (CaSki, HeLa and SiHa) were treated with control siRNA or G9a siRNA for 24 hrs or were treated with BIX01294 for 4 hrs. After washing out the medium, cells were incubated in a fresh culture medium for 24 hrs to collect total cell lysate, which was used for apoptosis-related protein (cIAP-1, HSP70 and XIAP) determination by western blot. Level of GAPDH was used as a loading control.

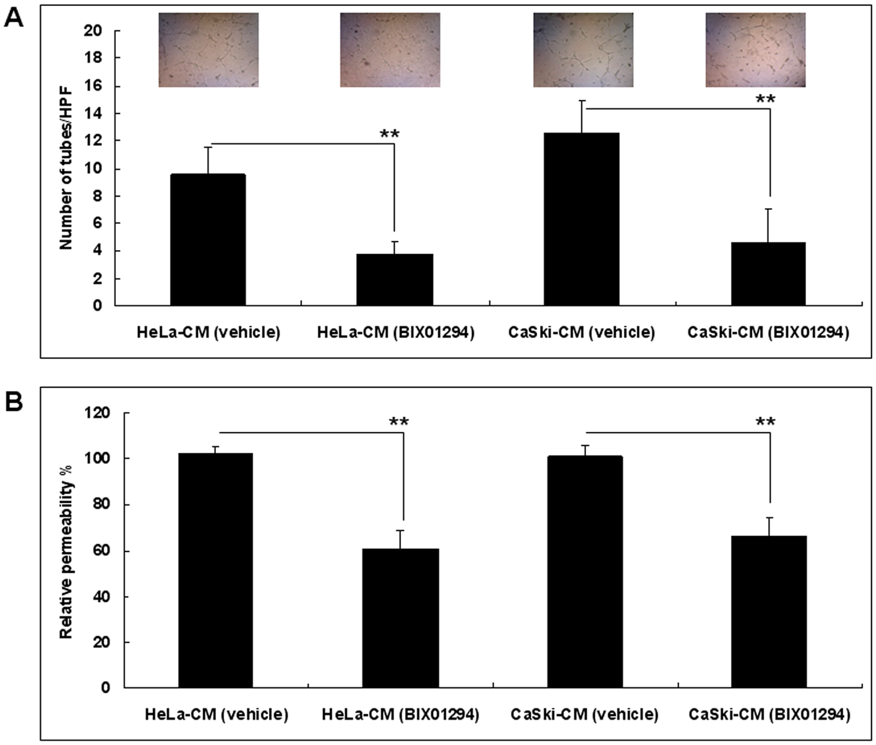

Supplementary Figure 11: Confirmation of the effect of G9a chemical inhibitor BIX01294 on angiogenesis. Cervical cancer cells (CaSki and HeLa) were treated with vehicle (5 µL saline) or 5 µM of BIX01294 for 4 hrs; after washing out the medium, cells were incubated in fresh culture medium for 24 hrs to collect conditioned medium, and then used for **(A)** an endothelial cell tube formation assay. Data were the number of polygonal vascular tube formations per HPF (100x) under different conditions. $n = 5$. $^{**}p < 0.01$. Data are presented as mean ± SD. **(B)** An endothelial cell permeability assay. Data were relative permeability, in which vehicle-treated conditioned medium is defined as 100%. $n = 5$. $^{**}p < 0.01$. HPF: high power field. CM (vehicle): conditioned medium from vehicle-treated cells. CM (BIX01294): conditioned medium from BIX01294-treated cells.

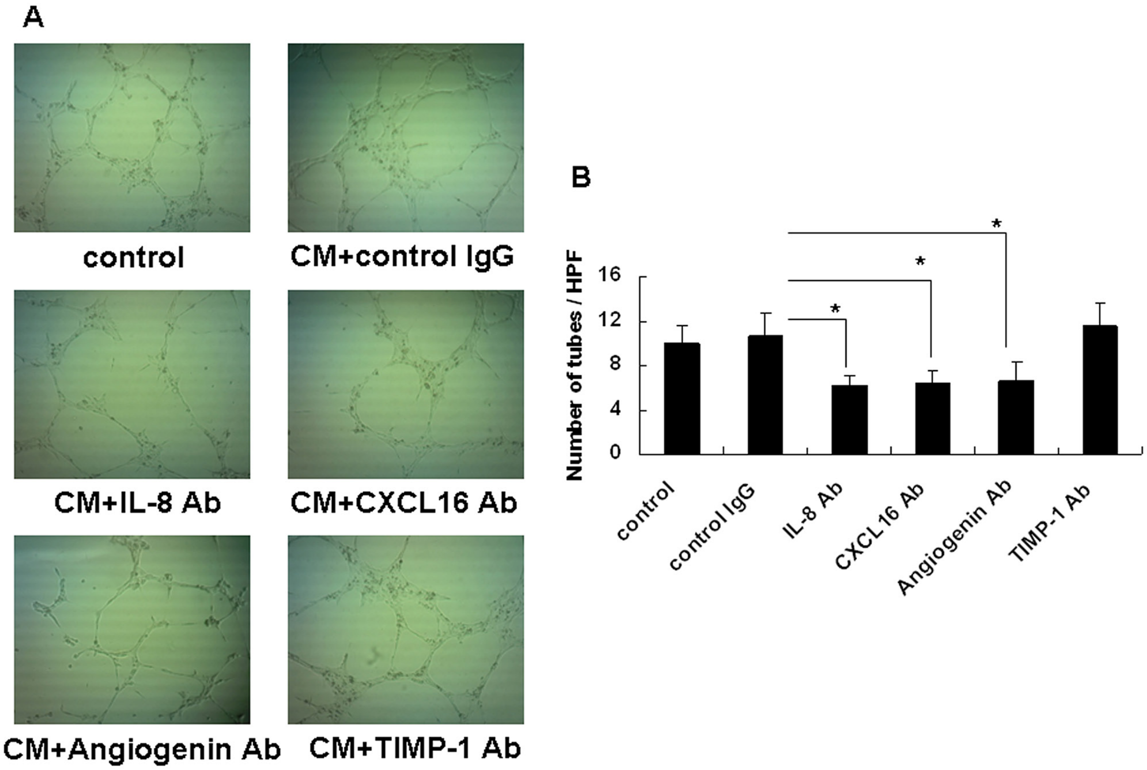

Supplementary Figure 12: Confirmation of the angiogenesis-promoting capabilities of interleukin-8, CXCL16, angiogenin and TIMP-1. SiHa cells were incubated in fresh culture medium for 24 hrs to collect the conditioned medium. Conditioned media were incubated with interleukin-8, CXCL16, angiogenin and TIMP-1 neutralizing antibodies (2 μg/ml) for 30 min and then used for an **(A)** endothelial cell tube formation assay (100x). **(B)** Data from (A) were the number of polygonal vascular tube formations per HPF (100x) under different conditions. $n = 5$. $*p < 0.05$. Ab: antibody. CM: conditioned medium. HPF: high power field. IL-8: interleukin-8.

Supplementary Table 1: Apoptosis array data

Spot	Name	Mean	SD
A1 2	Reference spots	0.993	0.100
A23 24	Reference spots	1.015	0.124
B3 4	Bad	0.947	0.072
B5 6	Bax	1.073	0.093
B9 10	Pro-caspase-3	0.957	0.098
B11 12	Catalase	0.976	0.104
B15 16	cIAP-1	0.952	0.132
B19 20	Claspin	0.954	0.130
B21 22	Clusterin	0.885	0.121
B23 24	Cytochrome c	1.054	0.154
C1 2	TRAIL R1/DR4	1.012	0.121
C3 4	TRAIL R2/DR5	1.057	0.114
C5 6	DAFD	0.961	0.100
C7 8	Fas/TNFRSF6/CD95	0.935	0.112
C9 10	HIF-1alpha	1.002	0.201
C11 12	HO-1/HMOX1/HSP32	0.960	0.159
C13 14	HO-2/HMOX2	0.812	0.136
C15 16	HSP27	0.940	0.133
C17 18	HSP60	0.879	0.154
C19 20	HSP70	1.057	0.158
C21 22	HTRA2/0mi	0.861	0.134
D15 16	SMAC/Diablo	0.977	0.141
D17 18	Survivin	1.009	0.156
D21 22	XIAP	0.880	0.137
E1 2	Reference spots	1.014	0.093

Supplementary Table 2: Angiogenesis array data

Spot	Name	Mean	SD
A1 2	Reference spots	0.969	0.095
A17 18	Angiogenin	0.207	0.192
A19 20	Amphiregulin	0.566	0.140
A23 24	Reference spots	0.997	0.110
B1 2	Coagulation factor III	0.737	0.126
B3 4	CXCL16	0.281	0.108
B5 6	DPPIV	2.297	0.115
B15 16	Endothelin-1	2.436	0.080
C3 4	GM-CSF	0.624	0.096
C13 14	IGFBP-3	0.930	0.123
C17 18	IL-8	0.151	0.107
C23 24	MCP-1	0.739	0.096
D5 6	MMP-9	0.783	0.135
D9 10	Pentraxin 3	0.863	0.091
D13 14	PDGF-AA	0.898	0.106
D17 18	Persephin	1.078	0.191
E3 4	Serpin E1	0.631	0.084
E7 8	TIMP-1	1.021	0.107
E9 10	TIMP-4	0.528	0.156
E11 12	Thrombospondin-1	1.042	0.157
E19 20	VEGF	0.970	0.148
F1 2	Reference spots	1.130	0.084

Supplementary Table 3: Case distribution of G9a expression in deceased and surviving patients*

G9a expression	0	1	2	3	Total
Deceased	2 (7.4%)	3 (11.1%)	11 (40.7%)	11 (40.7%)	27 (100%)
Surviving	6 (21.4%)	7 (25.0%)	10 (35.7%)	5 (17.9%)	28 (100%)
Total	8 (14.5%)	10 (18.2%)	21 (38.2%)	16 (29.1%)	55 (100%)

*p-value <0.05. Chi-square test for trend.
